# Recent advances in enzymatic biofuel cells enabled by innovative materials and techniques

**DOI:** 10.1002/EXP.20220145

**Published:** 2023-06-01

**Authors:** Wengang Huang, Muhammad Yazid Bin Zulkifli, Milton Chai, Rijia Lin, Jingjing Wang, Yuelei Chen, Vicki Chen, Jingwei Hou

**Affiliations:** ^1^ School of Chemical Engineering The University of Queensland Saint Lucia Queensland Australia; ^2^ School of Chemical Engineering The University of New South Wales Sydney New South Wales Australia; ^3^ Australian Institute for Bioengineering and Nanotechnology The University of Queensland Saint Lucia Queensland Australia

**Keywords:** bioelectrodes, biosensors, enzymatic biofuel cells, enzyme cascade, implanted devices, metal‐organic frameworks, single enzyme biofuel cells

## Abstract

The past few decades have seen increasingly rapid advances in the field of sustainable energy technologies. As a new bio‐ and eco‐friendly energy source, enzymatic biofuel cells (EBFCs) have garnered significant research interest due to their capacity to power implantable bioelectronics, portable devices, and biosensors by utilizing biomass as fuel under mild circumstances. Nonetheless, numerous obstacles impeded the commercialization of EBFCs, including their relatively modest power output and poor long‐term stability of enzymes. To depict the current progress of EBFC and address the challenges it faces, this review traces back the evolution of EBFC and focuses on contemporary advances such as newly emerged multi or single enzyme systems, various porous framework‐enzyme composites techniques, and innovative applications. Besides emphasizing current achievements in this field, from our perspective part we also introduced novel electrode and cell design for highly effective EBFC fabrication. We believe this review will assist readers in comprehending the basic research and applications of EBFCs as well as potentially spark interdisciplinary collaboration for addressing the pressing issues in this field.

## Graphical abstract

Enzymatic biofuel cells (EBFCs) have garnered significant research interest due to their capacity to power implantable bioelectronics, portable devices, and biosensors by utilizing biomass as fuel. To depict the current progress of EBFC and address the challenges it faces, this review traces back the evolution of EBFC and focuses on contemporary advances.

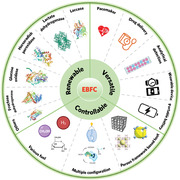

## 1 INTRODUCTION

**FIGURE 1:**
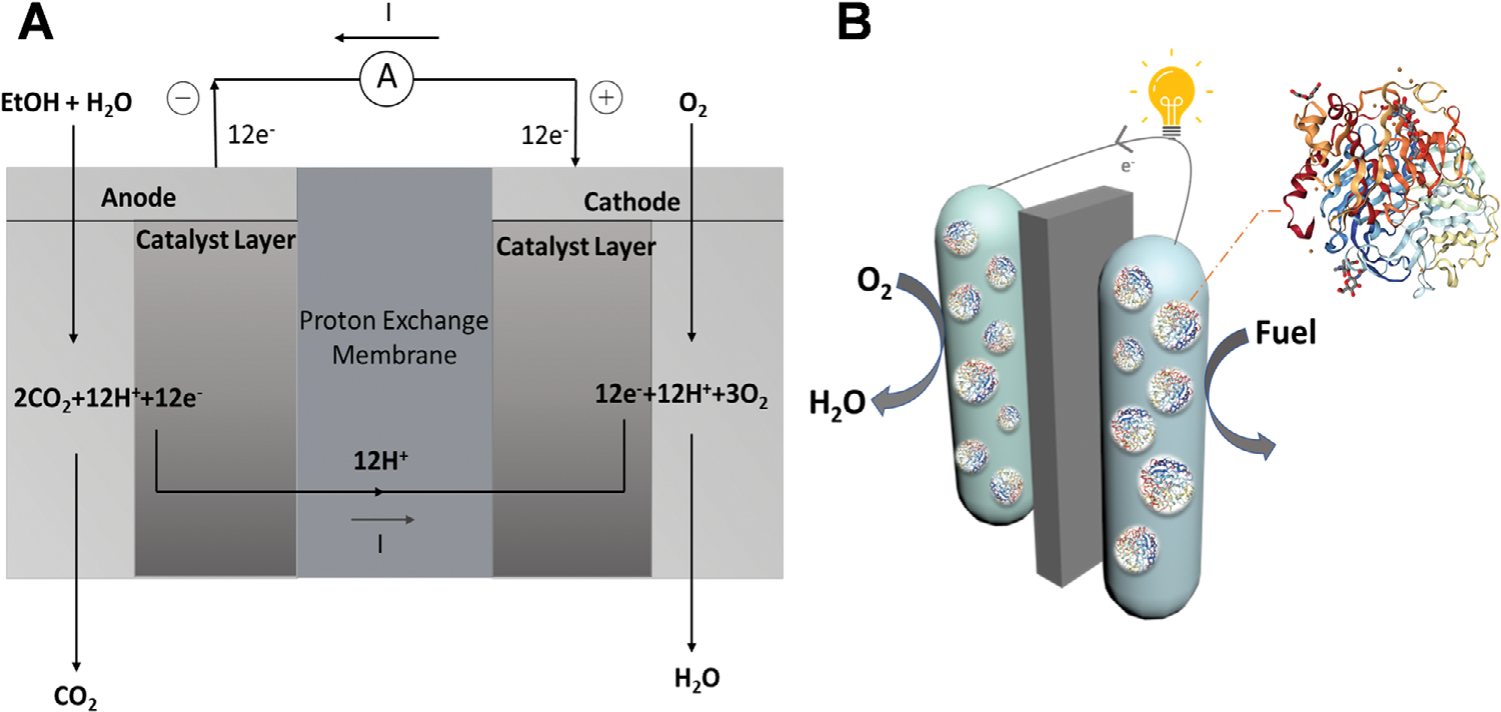
(A) Schematic of ethanol (EtOH)/O_2_ fuel cell setup. (B) Schematic diagram of enzymatic biofuel cell (EBFC).

The limited supply of fossil fuel to meet the ever‐increasing energy consumption, coupled with the severe environmental consequences of fossil fuel combustion, necessitates the pursuit of an alternative green and renewable energy source. On account of recent trends toward net‐zero carbon emission, fuel cell systems are viewed as promising technologies that can generate clean energy directly from chemical fuels, unlike solar cells and wind power, which require conversion and additional storage processes. This enables greater deployment flexibility for fuel cells as renewable and alternative energy sources.^[^ ^1^ ^]^ Essentially, fuel cells are electronic devices that could convert chemical energy into electrical energy by utilizing noble metals as electrodes, as illustrated by a prototypical ethanol/oxygen fuel cell in Figure 1A. Electrons liberated at the anode by electro‐oxidation of pure fuels (e.g., hydrogen, ethanol, methanol) travel through the external circuit to the cathode, where they reduce an oxidant (e.g., O_2_).^[^ ^2^ ^]^ Conventional fuel cells have significant advantages over other energy conversion processes, including a wide operation temperature range (45°C–150°C) and stable energy output in harsh operating environments. However, some factors continue to limit their widespread application, such as the requirement of expensive and non‐renewable noble metal catalysts in electrodes, issues with electrode passivation at extreme pH levels, the need for membranes to separate reactions, and the demand for high purity fuels.^[^ ^3^ ^]^

**FIGURE 2:**
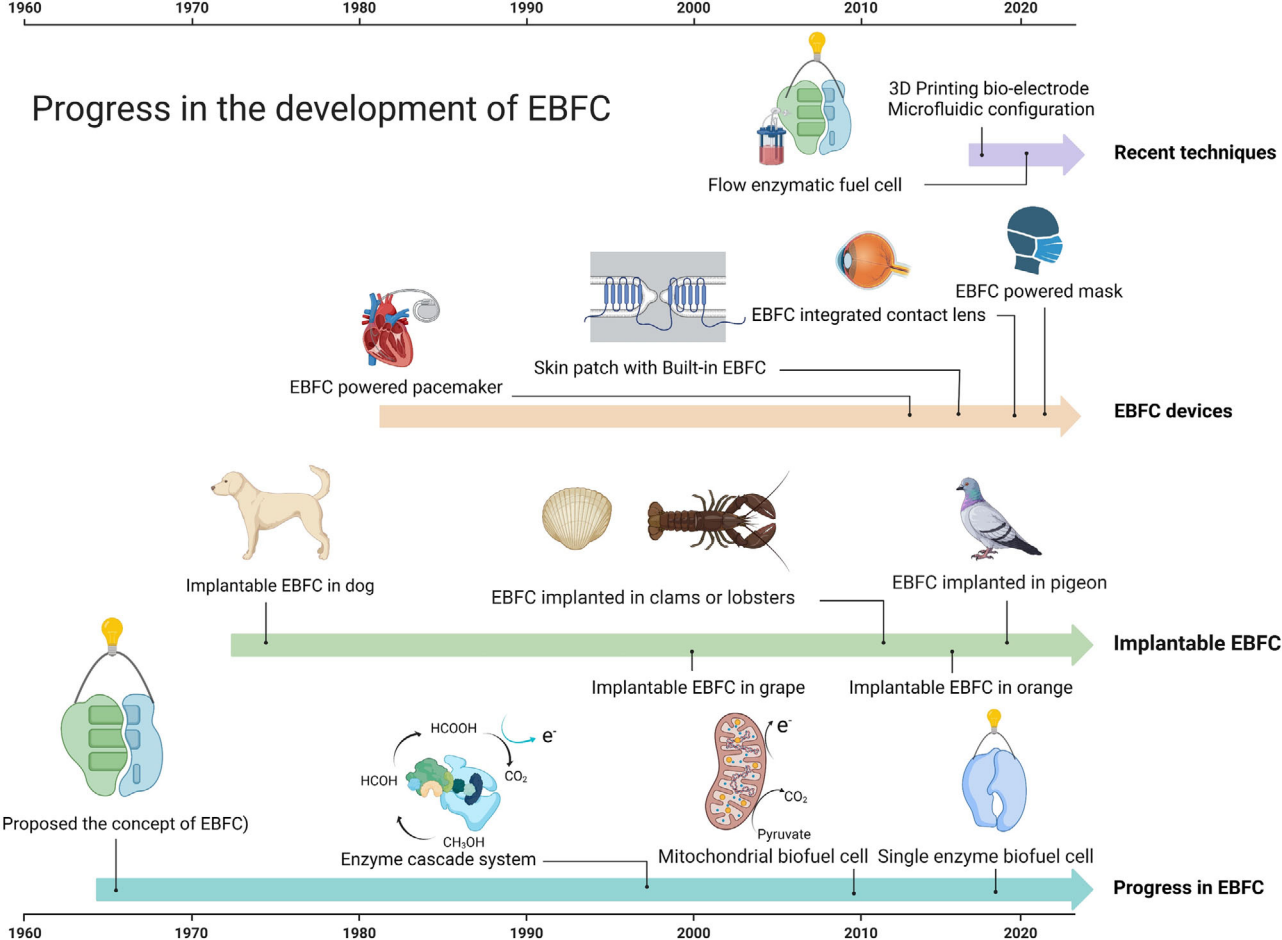
Progress in the development of enzymatic biofuel cell (EBFC) and applications. Figure created with BioRender.com.

Biofuel cells, which require enzymes or microorganisms as catalysts, are a subfamily of the fuel cell system that has constantly attracted research interests over the years as it provides a cost‐effective alternative to noble metal‐based electrodes.^[^ ^4^ ^]^ The concept of biofuel cell was first introduced in 1912, and has gradually developed into a research hotspot in the following decades. With the United States space program's interest in developing biofuel cells in the middle of 20th century, the practical impact of biofuel cells was improved dramatically.^[^ ^5^ ^]^ The long lifetime of microbial and its excellent oxidization ability of sugar to CO_2_ encouraged sustainable and economically feasible green energy generation. However, the low energy density and conversion efficiency of microbial biofuel cells limited its application and stimulated the development of next‐generation biofuel cell.^[^ ^6^ ^]^ Inspired by the unique biocatalysts property of enzymes, Yahiro et al. initially proposed enzymatic biofuel cell (EBFC) with an anode‐based on the glucose oxidase (GOx) enzyme.^[^ ^7^ ^]^ EBFCs (Figure 1B) exploit enzymes or enzyme complexes as biocatalysts to convert chemical energy to electrical energy by promoting the oxidation and reduction of fuel and oxidant (oxygen or peroxide). Notably, EBFCs expose catalytic sites for direct contact with fuel, which significantly increases catalytic efficiency. Therefore, the power output of EBFCs reached a relatively higher threshold (mW cm^−2^) in comparison with the initial level (µW cm^−2^), paving the way for practical low‐power applications.^[^ ^8^ ^]^ The improved catalytic activity of EBFC attributes to the enzyme's nano‐size and more highly specific reaction sites. For example, even a grape‐based EBFC is capable of producing 2.4 W of power at 0.52 V.^[^ ^9^ ^]^ In addition, the employment of enzymes, instead of cytotoxic microbial systems, prevented the leave of any hazardous heavy metals or bacterial toxins, making EBFCs a safe and reliable power source for implantable and wearable devices.^[^ ^10^ ^]^ Its properties have piqued the interest of an increasing number of scientists, and recent development progress is depicted in Figure 2.

There are several benefits of EBFCs over conventional noble metal fuel cells: Enzymes are clean and renewable biological catalysts that can be readily extracted from living organisms; Mild operating conditions as enzyme catalyzed reactions are typically conducted under physiological temperature and pH conditions; Wide range of enzyme sources and high specificity toward different types of substrates. These benefits make EBFCs a topic of interest leading to increasing publications over the years.^[^ ^11^ ^]^ However, several challenges still impede their real‐world applications, such as undesired immobilization density, incompatibility between cofactors and enzymes, the lower voltage output, and the operational stability concerns.

In this review, we not only covered some fundamental aspects of EBFC, such as enzymes (including the O_2_ sensitivity issue), fuels, and multi or newly emerging single enzyme configuration,^[^ ^12^ ^]^ but also advanced materials and technologies. Moreover, we also provide possible solutions to problems faced in EBFC.

## 2 FUNDAMENTAL PUZZLE—ENZYME AND FUEL FOR EBFCs

### 2.1 Configuration of enzymes in EBFCs

#### 2.1.1 Common enzymes for the anodic reaction

A typical anodic reaction for EBFCs works is conducted by employing enzymes to oxidize the fuel in the anode, the released electrons transfer to the cathode through the circuit, followed by hydrogen protons transfer through the interior circuit to complete the energy conversion. Currently, the widely explored enzymes in the anode include GOx, hydrogenases, and glucose dehydrogenase (GDH).

As an extensively studied biocatalyst for the anodic reaction, GOx is composed of two identical subunits, each with a molecular weight of about. 80 kDa, 8 nm in particle size.^[^ ^13^ ^]^ One subunit is responsible for binding of the substrate (β‐D‐glucose) while the other is in charge of the non‐covalently binding of flavin adenine dinucleotide (FAD), the redox cofactor for the catalytic function of GOx. GOx catalyzes the conversion of glucose to D‐glucono‐1,5‐lactone, which hydrolyses spontaneously to gluconic acid. Electrons are transferred from the reduced co‐enzyme (FADH_2_) to oxygen during this reaction. It should be noted that some enzymes like GOx or hydrogenase are quite sensitive to oxygen and may lead to electrons’ side reaction instead of their external circuit: the oxidation reaction's natural electron acceptor is oxygen, which sequentially reduces to hydrogen peroxide,^[^ ^13^ ^]^ this self‐consuming fuel process reduces EBFC's efficiency and energy density. Therefore, to achieve a higher power density, it is crucial to avoid the formation of hydrogen peroxide near the electrode, particularly in a single‐chamber system where oxygen directly competes with the anode for released electrons.

Regarding the oxygen sensitivity issue, here we consider another common enzyme of which their application has been severely hindered: hydrogenases. Hydrogenases are widely used in EBFC as anodic catalysts. They are efficient catalysts that promote the conversion of hydrogen, an environmentally friendly and economical chemical, to protons and electrons. Although oxygen can inhibit hydrogen oxidation, enzyme catalytic reaction was still active even at oxygen concentrations higher than those found in air, paving the way for the potential application of a hydrogenase‐based EBFC. In 2005, Armstrong and his colleagues reported the first use of pure hydrogenase in H_2_/O_2_ EBFC.^[^ ^14^ ^]^ Recently, Plumeré’s group proposed a possible solution for the oxygen sensitivity issue by applying novel homogeneous polymer thin films (viologen‐modified dendrimer films) that provided protection against O_2_ while achieving highly efficient enzymatic catalyst utilization.^[^ ^15^ ^]^ In 2020, a shielded gas diffusion bioanode was designed by integrating redox polymers in the bioanode for the protection of O_2_ sensitive hydrogenase enzyme, enabling operation under a low O_2_ concentration (O_2_ 5% / 95% Ar).^[^ ^16^ ^]^ These achievements are attributed to the redox polymer's ability to capacity to conduct electrons within the film. The polymer's electron charge transfer with hydrogenases as well as catalytic activity for O_2_ reduction allow for the partial diversion of electrons generated from H_2_ oxidation toward the reduction of the O_2_ molecules that penetrate the film at the matrix/solution interface, thereby protecting the immobilized hydrogenase enzymes from oxygen. However, a limited number of enzymes effectively contribute to the current generation in the EBFC, and result in restricted energy densities (≤ 0.2 mW cm^−2^).^[^ ^17^ ^]^

Alternately, the combination of O_2_‐tolerant hydrogenases from extremophilic bacteria with bioelectrodes for directing electron transport also led to an improvement in the power density of EBFCs.^[^ ^18^ ^]^ In 2016, Kano's group reported a H_2_/O_2_ EBFC in which the *P*_max_ can reach greater than 8 mW cm^−2^ at 0.7 V.^[^ ^19^ ^]^ However, there is no obvious data to show the output stability of their device. A more thorough investigation reveals that this strategy has the potential to outperform others. Based on a similar strategy, Mazurenko et al. established a stable and high‐power density hydrogenase‐based EBFC, in particular, the device's energy production can be maintained at higher than 15 mW h even over 17 h of uninterrupted operation. They also developed a computational model for this EBFC to gain more insight into the behavior of hydrogenase in a gas diffusion porous electrode, an optimal thickness, and geometry of the porous bioelectrode were also proposed.^[^ ^17a^ ^]^ Kulka‐Peschke et al. provide a deeper understanding of the mechanism of some O_2_‐tolerant hydrogenases operation, they found reversible combination of glutamate or other appropriate ligands, initiated by an off‐site redox stimulation, may provide the mechanism for preventing unintended interactions of catalytic metal sites([FeFe]or [NiFe] site) with O_2_.^[^ ^20^ ^]^ These improvements and discoveries confirm that hydrogenase‐based EBFCs can be significantly enhanced by future advanced materials engineering combined with bioengineering and unique design strategies for niche applications.

Given that GDH does not require oxygen as its natural electron acceptor, it possesses certain benefits over GOx. However, the requirement for several soluble co‐enzymes including nicotinamide adenine dinucleotide (NADH/NAD^+^), pyrroloquinoline quinone (PQQ), and FAD still limits its application as implantable EBFCs.^[^ ^21^ ^]^ Recently, Gorton has introduced cellobiose dehydrogenase (CDH), a potential enzyme for biofuel cell that can use lactose as fuel.^[^ ^22^ ^]^ This enzyme has a one‐of‐a‐kind structure that consists of a catalytically active FAD dehydrogenase domain and a heme b cytochrome domain linked by a peptide linker region. Choi et al. used cheese whey from dairy production to generate electron power in an EBFC system with CDH.^[^ ^23^ ^]^ Other dehydrogenases utilized in EBFCs include PQQ‐dependent GDH^[^ ^24^ ^]^ and fructose dehydrogenase (FDH)^[^ ^25^ ^]^ for the oxidation of glucose and fructose, respectively.

#### 2.1.2 Common enzymes for the cathodic reaction

After being oxidized by enzymes at the anode, the hydrogen protons and electrons from fuel travel to the cathode through the electrolyte and external circuit respectively, where they are consumed in the charge transfer reduction. Laccase and bilirubin oxidase (BOD) are two common oxygen‐reducing enzymes. These enzymes at the cathode will complete the energy production cycle by conducting reduction reactions. These enzymes have four copper centers for catalytic reactions, which are classified into three types: T1 oxidizes the sacrificial fuel, whereas T2 and T3 readily reduce O_2_ to water.^[^ ^26^ ^]^ Laccases are most active in moderately acidic environments and are typically employed at a pH of 5. Barrière et al. found the laccase‐modified cathode is very sensitive to pH and becomes inefficient close to physiological pH of 7.5, yielding only 7% of the catalytic current observed at pH 5.0.^[^ ^27^ ^]^ In contrast, BOD is active in relatively alkaline conditions, enabling utilization at neutral pH, which facilitates its application in implanted devices. Cytochrome oxidase is also a well‐investigated oxygen‐reducing enzyme that uses heme as the catalytic center.^[^ ^28^ ^]^ Wang et al. demonstrated a new electron transfer pathway from Cyt c4 to CcO. They also succeeded in integrating membrane proteins, which then directly exchange electrons with the electrode when incorporated in hydrophobic carbon nanofiber networks, opening up new avenues in the understanding of the catalytic mechanism of O_2_ reduction at low pH.^[^ ^29^ ^]^

As these enzymes catalyze the reduction of O_2_ at the cathode, it should be assembled with a gas diffusion layer to enable a high surface area of contact between the enzymes and oxygen, in a configuration similar to a membrane electrode assembly applied in conventional fuel cells. This configuration helps to improve the cathode's reaction rate, as it refers to the bio‐three‐phase interface: gaseous substrates diffuse into the thin liquid layer surrounding the immobilized enzyme by dissolving in it from the gas phase. When bioelectrocatalysis is conducted in a steady state, the gaseous substrate reacts with the enzyme immediately.^[^ ^30^ ^]^ However, it also imposes challenges from mechanical/chemical durability. For instance, an imperfect layer's exposure to the organic biofuel solution may cause its swelling and eventually result in cathode decomposition.^[^ ^12b^ ^]^ The activity of the immobilized enzyme is also greatly affected by changes in local pH at and around the bio‐three‐phase interface. A balance between hydrophilicity and hydrophobicity in the bio‐three‐phase interface should be achieved to improve stability.^[^ ^30^ ^]^

### 2.2 Fuels driving EBFC processes

**FIGURE 3:**
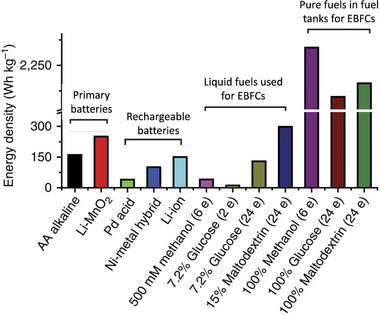
Comparison of energy densities among several batteries and enzymatic biofuel cells (EBFCs). The number of electrons involved is indicated. Reproduced with permission.^[^ ^32^ ^]^ Copyright 2014, Springer Nature.

Compared to conventional noble metal catalyzed fuel cells, the majority of which run on H_2_ or CH_3_OH, the fuel range of EBFCs has been greatly increased to include natural molecules commonly consumed by living beings, such as simple sugars (glucose, fructose, sucrose, and maltose) and alcohols (methanol, ethanol). Table 1 lists the fuels for some representative EBFCs together with their corresponding enzymes, electrode materials, and power output. The energy densities of EBFCs are relatively comparable to those of traditional primary and rechargeable batteries if fuels can be fully oxidized, as shown in Figure 3.

**TABLE 1:** Representative EBFC‐enabled bioelectronics and their parameters.

Electrode materials and their properties		Enzymes in electrodes	Fuel	Reaction in electrode	EBFC parameter	Highlight	Ref.
Carbon nanotubes (Good stability and conductivity)	CNTs composed of bucky paper	Laccase and glucose dehydrogenase	Glucose	Anode: glucose → glucono‐1,5‐lactone + 2H^+^ + 2e^−^ Cathode: O_2_ + 4H^+^ + 4e^−^ → 2H_2_O	*P*_max_: 800 mV, 25 µA, 5.2 µW for the serial connections of 3 implanted “electrified” living clams	EBFCs were implanted in living clams and produced long‐term electrical power in vivo	[34]
	CNT‐modified carbon fabrics	Bilirubin oxidase and fructose dehydrogenase	Fructose	Anode: fructose → 5‐dehydrofructose + 2H+ + 2e^−^ Cathode: O_2_ + 4H^+^ + 4e^−^ → 2H_2_O	OCV: 0.75 V; *P*_max_: 60 µW cm^−2^	A built‐in EBFC that allows for transdermal iontophoretic delivery of chemicals into human skin	[35]
	CNT/enzyme pellets	Laccase and glucose dehydrogenase	Glucose	See above	OCV:0.57 V *P*_max_:193.5 µW cm^−2^	The first time introducing EBFC which can generate enough electricity from a mammal's body	[36]
	MWCNTs and bacterial cellulose	Laccase	Bisphenol A	Anode: bisphenol A → Intermediates + Quinone+ 2H^+^ + 2e^−^ Cathode: O_2_ + 4H^+^ + 4e^−^ → 2H_2_O	OCV: 0.14 V, Power density: 1.897 W cm^−3^	Only required one type of enzyme for EBFC, showing that wastewater could be recycled and used as fuel	[37]
	MWCNTs modified cellulose nanofiber	Laccase and glucose dehydrogenase	Glucose	See above	OCV: 0.434 V; *P*_max_: 27 µW cm^−2^	Constructed flexible and eco‐friendly electrode for EBFC	[38]
Carbon nanosheets (Ease of modification, nanosized thickness reduced diffusion distance)	Graphene	Glucose oxidase and bilirubin oxidase	Glucose	Anode: glucose → glucono‐1,5‐lactone + 2H^+^ + 2e^−^ Cathode: O_2_ + 4H^+^ + 4e^−^ → 2H_2_O	*P*_max_: 24.3 ± 4 µW at 0.38 V OCV: 0.58 ± 0.05 V	First reported graphene nano sheets/enzyme composite as bio‐electrode	[39]
	Nano graphene platelets	Laccase and glucose oxidase	Glucose	See above	*P*_max_: 57.8 µW cm^−2^	The application of nano graphene shows the improvement charge transfer efficiency	[40]
	Reduced graphene	Glucose oxidase	Glucose	See above	Maximum current: 3.5 ± 0.02 mA cm^−2^)	A table current around 3.5 ± 0.02 mAcm^−2^ can be produced by electrode of which the area is only 0.07 cm^2^	[41]
Carbon nanoparticles (High area to volume ratio)	Mesoporous CNPs	Glucose oxidase and bilirubin oxidase	Glucose	See above	*P*_max_: 95 µW cm^−2^	First used mesoporous nanoparticles as electrode material to wire enzyme	[42]
	Carbon nanodots	Glucose oxidase and bilirubin oxidase	Glucose	See above	*P*_max_: 40.8 µW at 0.41 V OCV: 0.93 V	First reported use of carbon nanodots to enhance direct electron transfer	[43]
Conducting polymers (Good conductivity, large surface area, and efficient charge transfer rates)	PEI	Glucose oxidase	Glucose	See above	*P*_max_: 0.66 mW cm^−2^	The bond between polymers and enzymes plays an important role in preventing the enzyme's denaturation	[44]
	Os‐redox polymer	Glucose oxidase and bilirubin oxidase	Glucose	See above	2.4 µW at 0.52 V	The first time constructed EBFC in the grape	[9]
	PANI	Glucose oxidase	Glucose	See above	OCV: 0.78 V; *P*_max_: 1.12 mW cm^−2^	A yellow LED could be turned on by three of their assembled EBFC	[45]
	Conducting polymer‐functionalized gold electrode	Lactate dehydrogenase	Lactic acid	Anode: lactic acid → pyruvate Cathode: PEDOT + e^−^ →PEDOT_red_	OCV: 0.4 V; *P*_max_: 33.8 µW cm^−2^	A self‐powered “sense–act–treat” system	[46]
	Conducting polymer nanoparticles	Glucose oxidase	Glucose	See above	OCV: 0.48 ± 0.035 V; *P*_max_: 0.78 ± 0.034 mW cm^−2^	An improved lifetime and power density EBFC was achieved by using their conducting polymer nanoparticles	[47]
MOFs (Large surface area, high enzyme loading rate, and activity)	ZIF‐8 modified polyurethane nanofiber	Glucose oxidase and laccase	Glucose	See above	OCV: 0.35 V Power density: 1.09 W m^−3^ at 0.25 V	This flexible EBFC's output performance remains relatively steady after stretching and twisting	[48]
	Co‐hemin MOF/chitosan composite	Cellobiose dehydrogenase	Lactose	Anode: lactose → 4‐O‐(galactopyranosyl)‐glucono‐1,5‐lactone + 2H^+^ + 2e^−^ Cathode: N/A	High sensitivity as bio‐detector (response in 5 s)	Achieved an efficient lactose bio‐detector	[49]
	MAF‐7‐based composite electrode	Glucose oxidase and horseradish peroxidase	Glucose	Anode: glucose → H_2_O_2_ Cathode:2H^+^ + H_2_O_2_ + 2e^−^ → 2H_2_O	OCV: 0.34 V *P*_max_: 119 mW cm^−2^	Enzyme cascade was achieved in MAF‐7	[50]
	ZIF‐8 derivate carbon materials	Glucose oxidase	Glucose	See above	OCV: 0.63 V *P*_max_: 310 µW at 0.23 V	Exhibited great surface area, good conversion rates, and stability	[51]
	ZIF‐8 in situ growth on polyurethane nanofibers	Laccase	BPA	See above	Power density: 1.33 W m^−3^	Successfully fabricated a MOF‐based stretchable electrode	[52]

Abbreviations: BPA, bisphenol‐A; CNT, carbon nanotube; EBFC, enzymatic biofuel cell; LED, light‐emitting diodes; MOF, metal‐organic framework; MWCNTs, multi‐walled carbon nanotubes.

Although a wide variety of fuels can be utilized in EBFCs, the different application scenarios necessitate consideration. For instance, it was recently reported that hydrogen, a high‐quality fuel, can be completely utilized in an EBFC catalyzed by hydrogenases for the conversion of N_2_ to Chiral Amino Acids; however, the difficulty of safely storing and distributing H_2_ and the possibility of oxygen poisoning the enzyme limited its biotechnology applications.^[^ ^31^ ^]^ Some simple alcohols, such as methanol and ethanol, have a broader application prospect in enzymatic fuel cells owing to their productiveness and availability through biomass fermentation. However, their toxicity to mammalian cells precludes their use in implanted electro devices.

It is well‐worth noting that the popularity of glucose for EBFCs attributed to it is inexpensive, readily available, and negligible toxicity to humans. Its power density is also remarkably high: ideally, the heat combustion of glucose is nearly 15 MJ kg^−1^ which means it can release more than 3500 Ah kg^−1^ if it is completely converted to carbon dioxide and water, while a typical lithium‐ion battery exhibit around 40 Ah kg^−1^.^[^ ^32^ ^]^ In addition, the wide presence of glucose in the blood makes glucose‐based EBFCs suitable for implantable applications. These excellent properties further inspired continuous interest EBFC, as several sugars (xylose, fructose, a structural isomer of glucose, cellobiose, sucrose, and polysaccharides) and disaccharides (lactose), have been utilized in EBFCs.^[^ ^33^ ^]^

### 2.3 Integrate enzymes and fuels in EBFC

#### 2.3.1 Enzyme cascade

**FIGURE 4:**
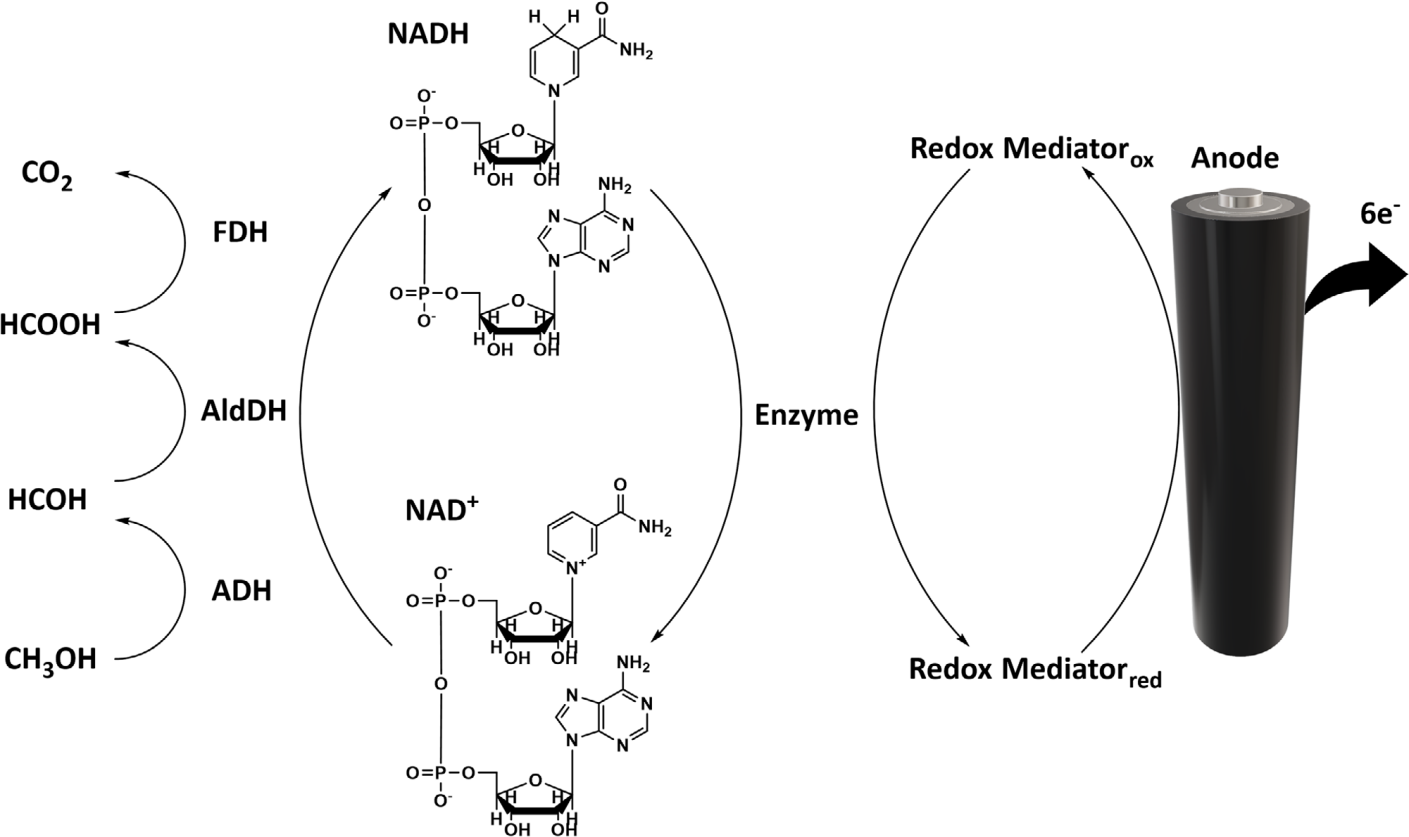
Cascaded oxidation of methanol coupled by cofactor regeneration system. Reproduced with permission.^[^ ^53^ ^]^ Copyright 1998, Elsevier. ADH, alcohol dehydrogenase; FDH, fructose dehydrogenase.

As described above, some biofuels’ heat combustion can achieve as higher as 15 MJ kg^−1^. However, the possibility of achieving the theoretical energy density in EBFC system is relatively low due to enzyme's high selectivity toward a one‐step reaction, only partial oxidation of fuel can be achieved in a single enzyme configuration. Hence, fuels must be deeply or completely oxidized by enzymes to improve power density: the enzyme cascade system (Figure 4) employs a series of enzymes at the anode to completely oxidize fuel, which was first proposed by Palmore et al. in 1998.^[^ ^53^ ^]^

**FIGURE 5:**
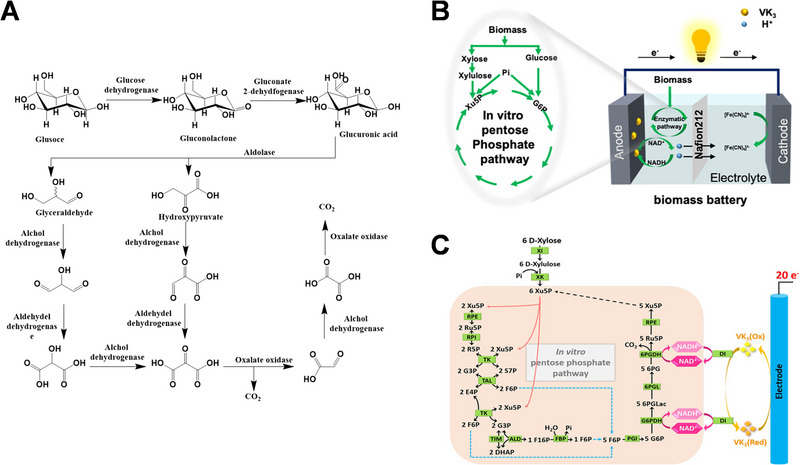
(A) Schematic of enzyme cascade oxidation pathway of glucose. Reproduced with permission.^[^ ^56^ ^]^ Copyright 2012, American Chemical Society. (B) Complete oxidization glucose and xylose pathway. Reproduced with permission.^[^ ^57^ ^]^ Copyright 2022, Elsevier. (C) Schematic of enzyme cascade oxidation of xylose. Reproduced with permission.^[^ ^58^ ^]^ Copyright 2018, Wiley‐VCH.

Early enzyme cascade systems utilized free enzymes while more recent research has focused on immobilizing the cascade to extend their operational lives and optimize performance by building molecular‐level approximations to stimulate cascade reactions.^[^ ^54^ ^]^ These advances triggered the exertion of glycerol as a fuel for a cascade system that adopts promiscuous PQQ‐dependent alcohol dehydrogenase, PQQ‐dependent aldehyde dehydrogenase, and oxalate oxidase to completely oxidize glycerol.^[^ ^55^ ^]^ This cascade system could be further extended with aldolase, GDH, and gluconate dehydrogenase to completely oxidize glucose via a parallel cascade (current density: 31.5 µA·cm ^−2^; power density: 6.74 µW·cm ^−2^),^[^ ^56^ ^]^ in which glucose is broken down into two intermediates that are oxidized in parallel, as shown in Figure 5A.

Very recently, Kizling et al. reported an EBFC with cascade enzyme configuration at the anode.^[^ ^59^ ^]^ In their work, many enzymes were utilized in conjunction with a single or mixed substrate. A comparison of previous studies demonstrates that the resultant bioanode is capable of producing a current greater than 2000 A cm^−2^ and sustaining power output for 8 days before falling to 38% of its initial level. Less prevalent sugars, such as xylose, can also be utilized as a fuel in cascaded EBFCs. Zhu's group designed a pathway for completely oxidizing xylose as schematically described in Figure 5B,C.^[^ ^57^, ^58^ ^]^ Around 14 enzymes were involved in this system and a high Faraday efficiency nearly 97% was achieved. Though better efficiency and power density can be achieved by cascaded EBFCs, the employ of enzyme cascades usually involves a complex reaction system, which causes several issues. Aside from the cascade system requiring a more sophisticated microstructure design to optimize the reaction kinetics, it is also difficult to find optimal operating conditions since different enzymes have different specificity in terms of temperature and pH values, limiting the overall system efficiency. Other concerns for the use of enzyme cascades for the EBFCs include the overall stability and spatial arrangement of the enzymes.^[^ ^60^ ^]^ Such obstacles can be tackled by either chemical (i.e., crosslinking or tethering) or biological (i.e., tethering) strategies (i.e., enzyme engineering).^[^ ^12a^ ^]^ Furthermore, electing nanoparticles with a high ratio of surface area to volume for enzyme loading minimizes the distance of electron transfer, hence enhancing the efficiency of cascaded EBFCs.^[^ ^59^, ^61^ ^]^

#### 2.3.2 Single enzyme biofuel cells

**FIGURE 6:**
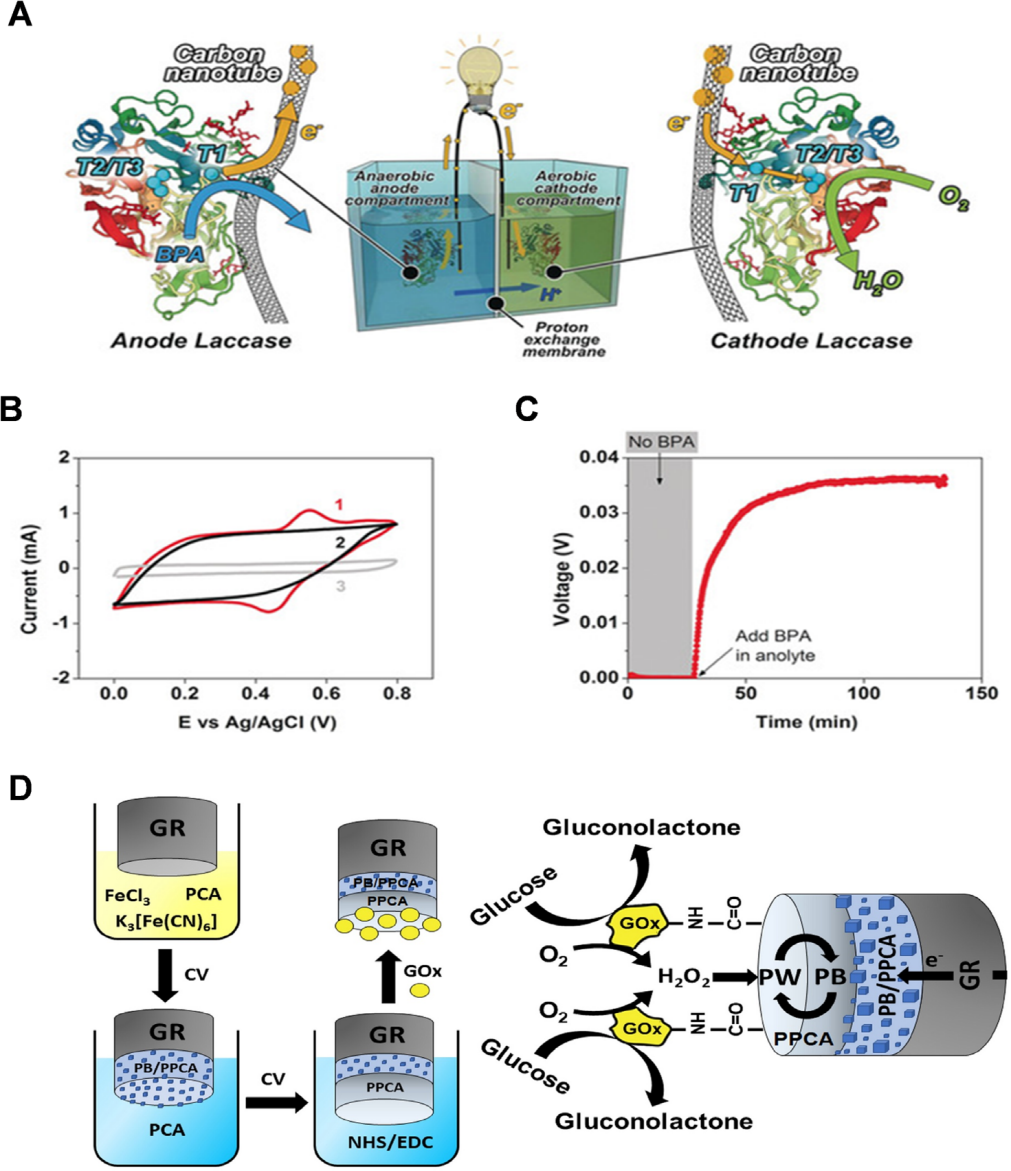
(A) Illustration of single enzyme biofuel cell powered by bisphenol‐A (BPA). Reproduced with permission.^[^ ^62^ ^]^ Copyright 2017, Wiley‐VCH. (B) CV of enzyme loaded carbon nanotube (CNT) electrodes (curve 1), CNT electrodes (curve 2), and benchmark (curve 3) electrodes at the same sweep rate. Reproduced with permission.^[^ ^62^ ^]^ Copyright 2017, Wiley‐VCH. (C) The working voltage of the cell. Reproduced with permission.^[^ ^62^ ^]^ Copyright 2017, Wiley‐VCH. (D) Fabrication of GR/PB‐PPCA/PPCA–glucose oxidase (GOx) biocathode. Reproduced with permission.^[^ ^64^ ^]^ Copyright 2021, Springer Nature.

In contrast to enzyme cascade, single enzyme biofuel cell, which was first reported by our group in 2017, is a novel biofuel cell concept that the same enzyme was applied in both the cathode and anode, and the reaction driving force was achieved through controlling the individual reaction conditions (mostly substrate concentrations) in different chambers, as shown in Figure 6A.^[^ ^62^ ^]^ The configure enables easier setup and optimization. For the benchmark system, laccase was the sole enzyme loaded at both electrodes in a combination of a two‐chamber fuel cell. Bisphenol‐A (BPA) was adopted as electron donor in the anolyte and oxygen as electron acceptor in the catholyte. Exceptionally, this configuration can actualize preferable energy generation in comparison to conventional EBFCs (e.g., working voltage: 0.12 V; *P*_max_: 160 mW m^−3^), at the same time, 98% of the BPA can be degraded enzymatically within the system after 12 h, transforming the organic pollutants into useful fuels. To further demonstrate the intramolecular electron‐harvesting notion at the anode, an enzyme‐based electrodes was established, producing enhanced anodic current commencing at around +0.70 V (vs. NHE) when BPA (0.05 mm) was introduced as shown in Figure 6B,C. The redox process at the T1 Cu site elucidates this phenomenon, indicating that electrons generated by substrate oxidation at the T1 Cu site can be transported to the electrode. Furthermore, the efficiency of this configuration can be optimized through electrode modification. Li et al. later reported a similar configuration by integrating carbon nanotubes (CNTs) with high conductivity and superior structural properties of bacterial cellulose (BC) as the backbone to entrap laccase.^[^ ^37^ ^]^ The resultant fuel cells achieved an open circuit voltage of 0.14 V and a power density as high as 1.897 W m^−3^. Li et al. also utilized laccase to create a single enzyme system‐based biosensor. Their maximum power density demonstrated an outstanding linear dynamic range from 0.01 to 0.4 mm with an excellent sensitivity for pollutant detection.^[^ ^63^ ^]^ Single enzyme system is also quite flexible for device fabrication, from the latest report, this system can be integrated on stretchable electrode, paving the way for the development of simple wearable electronics.^[^ ^52^ ^]^

Recently, Kausaite‑Minkstimiene et al. also introduced a glucose‐powered single enzyme biofuel cell.^[^ ^64^ ^]^ In their study, a modified graphite rod (GR) cathode comprised of a composite of Prussian blue (PB) and GOx was developed as shown in Figure 6D, possessing the advantages of low cost and reduced complexity of the system.^[^ ^64^, ^65^ ^]^ Though the power outputs of these single‐enzyme biofuel cells are still modest when compared to conventional EBFCs (nearly 149 W m^−3^, reported by Lang Xu and Fraser A. Armstrong^[^ ^66^ ^]^), this system can provide us with a more straightforward understanding of enzymatic reaction mechanisms since it proved that intramolecular electron transfer within individual enzyme molecules is an alternative avenue for power generation.

## 3 STRATEGIES FOR IMPROVING EBFC's PERFORMANCE

### 3.1 Coenzyme regeneration to promote EBFCs

**FIGURE 7:**
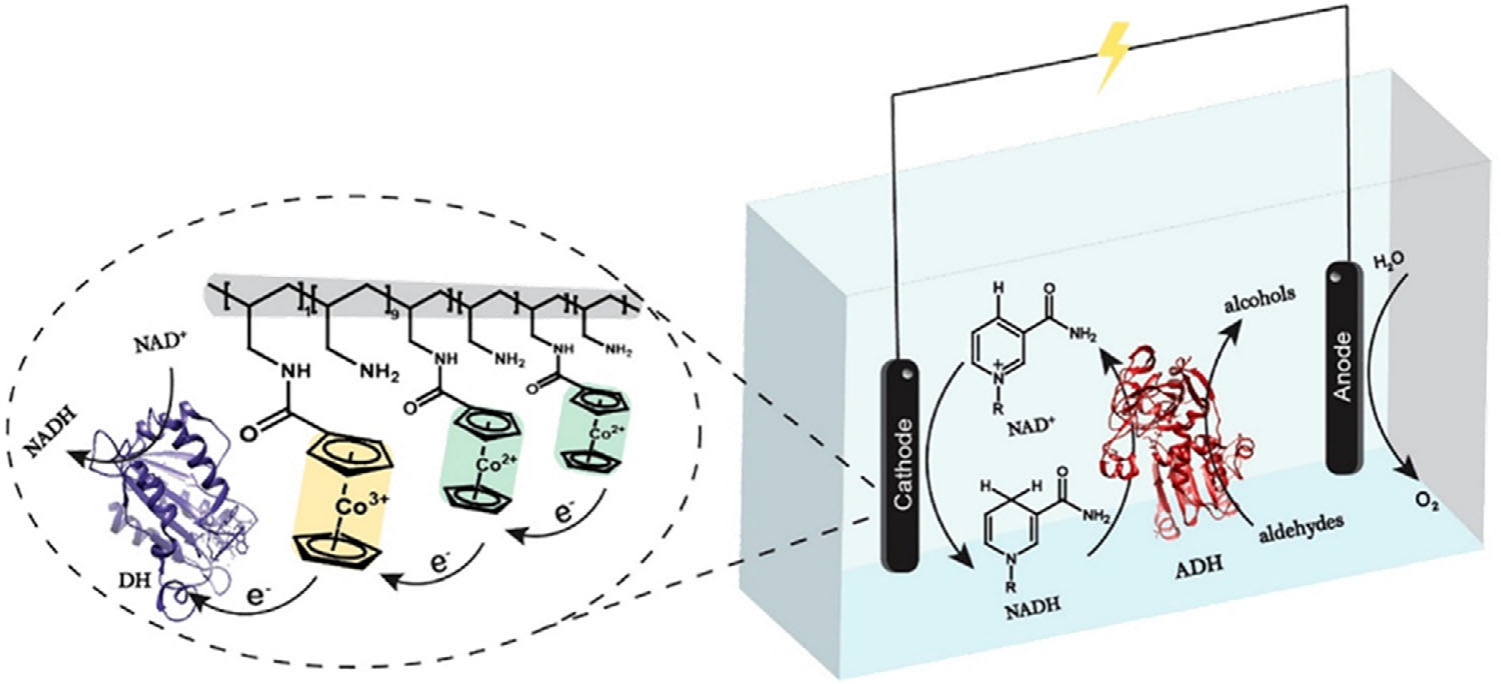
NADH production system coupled with alcohol dehydrogenase for electric production Reproduced with permission.^[^ ^69^ ^]^ Copyright 2019, American Chemical Society. ADH, alcohol dehydrogenase; DH, diaphorase.

Oxidoreductase enzymes often require the presence of redox equivalents (coenzyme/cofactor) that acts as a counterpart during the conversion of substrates.^[^ ^67^ ^]^ These coenzymes are generally expensive since they are almost exclusively bound to their natural coenzyme so they cannot be replaced by less costly substitutes unless they are altered through enzyme engineering.^[^ ^68^ ^]^ Therefore, the regeneration of coenzyme is critical to the economic viability of EBFCs utilizing oxidoreductases. An established method for the regeneration of a coenzyme is to utilize a cheap “sacrificial” substance to convert a coenzyme back to its original reduced/oxidized form (As shown in Figure 7, regenerated NADH can be consumed by ADH, regenerated by DH). From the aspects of economy and reliability, once this regeneration system is established it should be able to be implemented in a “plug‐and‐play” style for every EBFC system that requires a certain coenzyme.

Some enzymes and their coenzymes were listed in Section 2.1, for instance, nicotinamide adenine dinucleotide (phosphate) [NAD(P)H], NAD^+^, oxidoreductase cofactors like FAD, PQQ, hemes, iron‐sulfur clusters, coenzyme F420, flavin mononucleotide (FMN) and ascorbic acid. The most established and common cofactor for oxidoreductase enzymes is NAD(P), whose main function is the interaction between its oxidized (NAD^+^; NADP^+^) and reduced (NADH; NADPH) forms as a reduction/oxidation equivalent in redox reactions.

Since the reactions catalyzed by NAD(P)‐dependent enzymes are reversible, after converting a “sacrificial” substrate or fuel to an oxidation state, the regeneration reaction can be completed in a single step, yielding NAD(P)H. Inspired by this, very recently, Dr. Seong‐Min Jo et al. achieved an NAD‐regeneration reactor that only relies on O_2_ and H_2_O.^[^ ^70^ ^]^ This reactor has the potential for achieving artificial mitochondria, which might exhibit great potential for biocatalytic reaction application.

Several NAD(P)‐dependent dehydrogenases have been applied as anodes in EBFCs, including GDH,^[^ ^71^ ^]^ aldehyde dehydrogenase,^[^ ^72^ ^]^ alcohol dehydrogenase,^[^ ^73^ ^]^ malate dehydrogenase,^[^ ^74^ ^]^ formate dehydrogenase,^[^ ^53^ ^]^ pyruvate dehydrogenase,^[^ ^75^ ^]^ and lactate dehydrogenase.^[^ ^76^ ^]^ The most common enzymes used for NAD(P)H related regeneration are formate dehydrogenase (FDH, formate/ CO_2_) for the reduction of NAD^+^, and GDH (glucose/gluconolactone) for the reduction of NADP^+^.^[^ ^77^ ^]^ Notably, NAD‐dependent bioanodes are not dependent on dissolved oxygen as their final electron acceptors, they circumvent the sluggish oxygen dissolution kinetic terms, despite the fact that these regeneration systems are quite complex due to the difficulty of controlling the kinetics and optimizing the reaction conditions. The versatility of the cofactor configuration enables the usage of other types of dehydrogenases in biofuel cells, such as heme‐containing dehydrogenases, PQQ‐dependent dehydrogenases, and FAD‐dependent dehydrogenases. The reaction involving these enzymes are also oxygen‐independent, thus they are found to be of great research interest recently.

The most essential parts for coenzyme regeneration are reliable enzyme systems/electron pathways and proper materials for biomass immobilization. There are still many elusive questions that need to be addressed when integrating the co‐factor regeneration system with the EBFC setup. For example, which cofactor, NADPH or NADH, is the best to be regenerated? What are the best reaction conditions for cofactor regeneration, and would these conditions fit the reaction conditions for the enzymes in EBFCs? Could the cofactor generation process be integrated with the EBFC to generate an enzymatic cascade? The answers to these questions would shed light on promoting the reaction kinetics for the cofactor involved EBFCs.

### 3.2 Porous framework for enzyme immobilization

**FIGURE 8:**
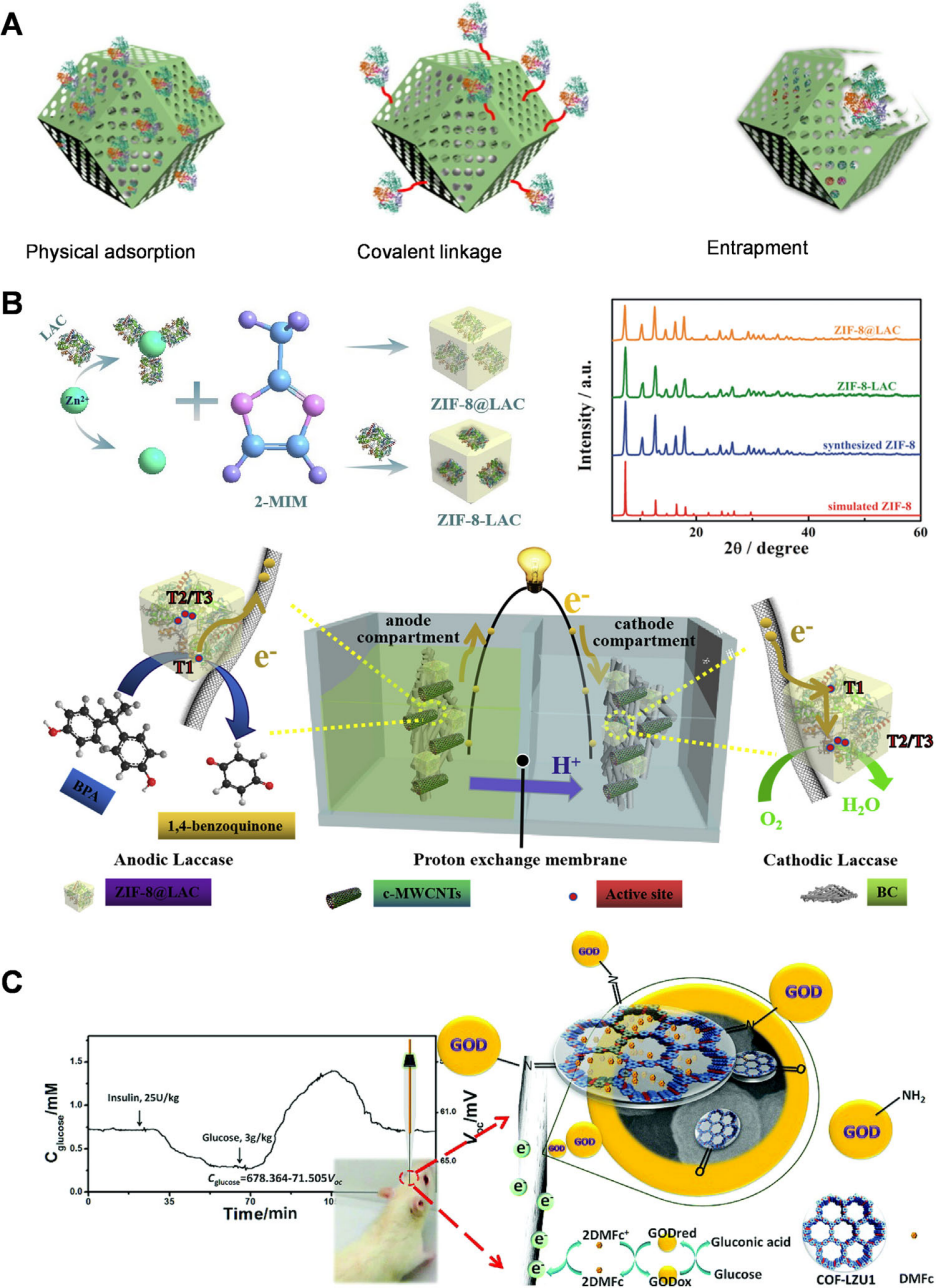
(A) Three strategies for enzyme immobilization in porous framework. Reproduced with permission.^[^ ^94^ ^]^ Copyright 2022, Elsevier. (B) Laccase entrapped in ZIF‐8 for single enzyme biofuel cell. Reproduced with permission.^[^ ^63^ ^]^ Copyright 2019, Elsevier. (C) COF‐enzyme biosensor for electrochemical measurements. Reproduced with permission.^[^ ^95^ ^]^ Copyright 2019, American Chemical Society.

The efficient immobilization of enzymes is critical in the fabrication of EBFC electrodes. It involves the use of substrates to anchor biocatalysts with a higher rate of interfacial energy transfer. These procedures are critical in ensuring the enzyme's catalytic performance, stability, properties, and reusability. It should be noted that a number of comprehensive review articles on enzyme immobilization methods are already available; therefore, our contribution will primarily focus on recent advancements in the field of porous framework materials, specifically metal‐organic frameworks (MOFs) and covalent‐organic frameworks (COFs), and discuss three methods for enzyme immobilization, as depicted in Figure 8A.

#### 3.2.1 Physical adsorption for porous framework

One of the most extensively used methods for enzyme immobilization is physical adsorption, which is a simple, versatile, and reversible approach that relies on van der Waals forces, hydrophobic contacts, or hydrogen bonds between the enzyme and the substrate material. It can largely remain the enzyme's pristine conformation and catalytic core unchanged. It is crucial in sustaining a wide spectrum of enzyme activity to the greatest extent possible under moderate settings. Recently, researchers found that MOFs and COFs are useful supporting matrices for enzyme adsorption or surface attachment due to their large surface area, simplicity of pore size tuning, ease of modification, and moderate synthetic conditions.^[^ ^78^ ^]^ Huang and colleagues demonstrated a unique porcine pancreatic lipase(PPL)@MOF bioreactor using microporous MOFs such as UiO‐66, UiO‐66‐NH_2_, and MIL‐53 for enzyme adsorption without any chemical modifications to the surface or macromolecule of the enzyme.^[^ ^79^ ^]^ In this work, the storage period and catalytic stability of PPL@MOFs generated in batches were also investigated. After 35 days of storage at 4°C, the PPL@MOFs demonstrated no discernible enzyme activity loss; it also demonstrated highly repeatable catalysis ability: PPL@MOFs prepared in three batches yielded a product formation with a relative standard deviation of less than 3%. In addition, the adsorption performance of HKUST‐1 and MIL‐100‐Fe are also investigated by other groups.^[^ ^80^ ^]^ Their straightforward method also demonstrated successful enzyme immobilization onto MOF surfaces, as well as high enzyme activity. In 2015, Sharath Kandambeth et al. reported a mesoporous COF with a high surface area.^[^ ^81^ ^]^ By adsorption method, it's enzyme storage capacity can achieve higher than 15 µmol g^−1^. Zhao et al. utilized COF to adsorb enzyme and discovered that, in comparison to conventional porous materials such as carbon or silica, their COF displayed superior adsorption due to its desirable pore size and electrostatic interaction.^[^ ^82^ ^]^ In addition, they confirmed that certain adsorbed enzymes are confined within the micropores or channels of COFs, which further ensured the activity and stability of enzymes. However, it still should be noted that while physical adsorption minimizes damage to enzyme's reaction sites, the weak interaction between materials and enzymes will lead to eventual desorption and deactivation of the enzyme during long‐term use.^[^ ^80^, ^83^ ^]^ On the other hand, physical adsorption allows for the easy reuse of costly immobilization support materials (MOFs and COFs) by removing the denatured enzymes from the substrates and reloading them with fresh enzymes to restore the activity.

#### 3.2.2 Entrapment in porous framework

Encapsulation methods are a typical technique for stabilizing enzymes in a “safe” milieu by trapping the enzyme to a frame, or particularly co‐precipitating enzymes within materials.^[^ ^84^ ^]^ Enzymes immobilized within the pores of hydrophobically modified micellar polymers including cellulose, nylon, polysulfone, collagen, polyacrylate acetate, and polycarbonate, for example, have been found to efficiently increase enzyme stability at electrode surfaces and extend their lifetime.^[^ ^84a^ ^]^ In addition to the above materials, MOFs and COFs are also currently being reported to entrap enzymes with comparable long lifetimes and increased biological activities.^[^ ^85^ ^]^ MOFs and COFs offer numerous benefits over conventional carbon supports and silica sol‐gels. These benefits consist of diverse features deriving from their unique preparation processes, making them excellent for enzyme encapsulation, and ease of modification.^[^ ^86^ ^]^ When studying the behavior of MOFs in EBFCs, attention should be paid to electron transfer between trapped biomaterials and electrodes. Zhong et al. created a MOF‐derived electrocatalyst for oxygen reduction reactions in biofuel cells by combining Co–Nx active components and employing Zr‐based MOF UiO‐66‐NH_2_. The BET surface area of this material remained even with the encapsulated biomass and had a more positive half‐wave potential (35 mV) than the benchmarked Pt/C substrates.^[^ ^87^ ^]^ The biofuel cell device, however, attained an OCV of 0.39 V and a maximum power density of 299.62 mW cm^−2^, which are still significantly lower than that obtained by commercial Pt/C benchmark electrodes. In light of this, it is important to consider how to compensate for the material's limitations. For example, the highly porous nature of MOFs and COFs usually leads to their poor electronic conductivity. In order to improve the conductivity of the framework‐enzyme electrode, one method discovered by a recent study was to add a phosphate buffer to boost the solution conductivity.^[^ ^88^ ^]^ However, given the likely solubilization behavior of MOF in such protonic solutions, the enzyme may be exposed to the external environment, resulting in denaturation or leaching from the substrates. An alternative approach to improve conductivity requires support substrates such as hydromagnesite flakes, melamine sponges, and carbon nanotubes to inherently hybridize the MOF structure.^[^ ^89^ ^]^ Wang et al. created an N–doped MOF by utilizing the isoreticular metal‐organic framework‐3 (IRMOF‐3) combined with carbon nitride.^[^ ^90^ ^]^ Their biofuel cell successfully exhibited an optimized oxygen reduction reaction (ORR) performance. Apart from considerations of the material conductivity, the pore size of the MOF or COF electrode for enzyme entrapment can also have an impact on EBFC's performance. A pore size that is too large can lead to enzyme desorption, while a pore size that is too small can result in lower mass transfer rates and even clogging of the pores.^[^ ^83^, ^91^ ^]^ Li et al. applied a zeolitic imidazolate framework‐8 (ZIF‐8) framework with a sufficient pore size to encapsulate laccase in their system as shown in Figure 8B, achieving a single enzyme biofuel cell and a self‐powered sensor for BPA detection.^[^ ^63^ ^]^ Finally, they coupled it with bacterial cellulose and carboxylated multi‐walled carbon nanotubes (c‐MWCNTs) to create a flexible electrode with significant biosensor potential. The enzyme cascade system also can be achieved in a MOF composite.

Entrapment can improve enzyme stability while also providing a microenvironment that lowers the possibility of interference with the enzyme, avoiding denaturation.^[^ ^92^ ^]^ This approach, when combined with conductive materials as described above, creates an excellent milieu for enzymes to perform biocatalysis and aid in electron transfer. Moreover, the ideal entrapment microenvironment, which is also dependent on the material substrate, contains optimal pH, temperature, and polarity, in which enzymes work or be encapsulated optimally.^[^ ^93^ ^]^

#### 3.2.3 Covalent bonding on porous frameworks

Covalent bonding is an irreversible direct covalent link formed by functional groups between enzymes and the supporting matrix. This technique typically involves modifying or functionalizing electrode surfaces, enabling covalent binding between the nucleophilic groups of the enzymes and the modified surface. It helps to maintain the enzyme's stability while also maintaining power density. Undoubtedly, covalent linking of enzymes to the surface of MOFs and COFs can significantly improve their stability. Park et al. pioneered this work by using carbodiimide to activate the carboxylate groups on the MOF surface. The activated carboxylates were then conjugated to additional protein pendent amino groups.^[^ ^96^ ^]^ Follow by this, Lou et al. also successfully immobilized soybean epoxide hydrolase on UiO‐66‐NH_2._^[^ ^97^ ^]^ Recently, Su et al. reported the utilization of COF to immobilize electron mediators and enzymes simultaneously.^[^ ^95^ ^]^ Their COF enzyme‐based biosensor exhibited high selectivity toward glucose and can be applied in vivo as shown in Figure 8C.

Covalent bonding is commonly employed to modify the surface of electrodes because it creates strong bonds between enzymes and the support matrix, resulting in low enzyme leakage from the support. Meanwhile, the amount of immobilization of the enzyme may be controlled. However, since the majority of enzymes must undergo chemical treatment to activate the functional group and this process requires a longer incubation period than adsorption, covalent bonding typically exhibits a significant risk of enzyme deactivation.^[^ ^98^ ^]^

### 3.3 Enzyme engineering

Enzyme engineering has emerged as a subfield for enhancing EBFCs.^[^ ^99^ ^]^ This strategy offers opportunities to enhance the efficiency of electron transfer between enzymes and electrodes, as well as the stability and overall performance of bioelectrode in EBFCs.^[^ ^100^ ^]^ More specifically, it allows the stability of the enzyme to be improved by altering the protein's secondary structure by introducing strong bonds, removing unwanted sites prone to degradation, and circumventing harmful steric effects.^[^ ^101^ ^]^

Enzyme subunits, for instance, have varied roles; by truncating certain subunits, enzymes can exhibit the desired performance. Zhu's group attempted to determine the role of four hydrogenase subunits.^[^ ^102^ ^]^ According to their report, an enhanced electron transfer rate between their hydrogenase and the electrode could be realized by truncating 2 subunits. Sode's group also observed an increased DET rate and the expected IET (intramolecular electron transfer) from artificial mutated enzymes.^[^ ^103^ ^]^ Moreover, altering subunits can also improve the enzyme's stability. According to a preliminary report of Sode's group, by substituting a single amino acid for cysteine, the half‐life of the EBFC‐utilized homodimeric PQQ GDH from Acinetobacter calcoaceticus was considerably increased from 52.4 h to 152 h. The enhanced stability is primarily attributed to the stabilization of the quaternary structure by a disulfide bond introduced in its dimer interface.^[^ ^104^ ^]^ Another relatively new method is the introduction of noncanonical amino acids into the target enzyme's designed sites. The ability to graft particular functional groups with chosen activities at a chosen sidechain of the enzyme, allowing for the rapid synthesis of conjugates via a covalent bonding between the sidechain and other chemicals or biomolecules, is a significant benefit of this approach.^[^ ^105^ ^]^ For instance, Guan et al. modified the tethering site of the enzyme by introducing the unnatural amino acid 4‐azido‐L‐phenylalanine (AzF), then the highly efficient copper‐free click chemistry reaction was exploited to connect the modified enzyme to electrodes.^[^ ^106^ ^]^ According to their investigation, the anchor of AzF had only a slight impact on enzyme kinetics. This also confirmed that optimizing a specific spot on the enzyme as an anchor point to the electrode can reduce activity/stability loss during immobilization and offer precise control of enzyme orientation on the electrode.

Additionally, efforts have been made to enhance the electro, thermal and chemical stability of enzymes. An improved enzyme system can be generated by directed evolution of enzyme. According to Zhu's report, a low pH tolerant enzyme was achieved by directed evolution.^[^ ^107^ ^]^ At pH 5.4, their modified enzyme system was 42 times more catalytically effective than the original enzyme. This strategy can also change enzyme's redox potential for enhanced electrochemical flexibility. Prof. Schwaneberg reported their directed evolution of a bacterial Laccase toward a more positive onset potential, leading to an enhanced power output of EBFC.^[^ ^108^ ^]^ Alternatively, enzyme's stability can be enhanced by inserting the connection into subunits of enzyme via the substitution of amino acids or directly employing extremophilic enzymes. Laccase from saccharomyces cerevisiae has been directly altered in the second coordination sphere of T1 to make it chloride ion resistant, making it more suitable for implantable EBFC applications.^[^ ^109^ ^]^ The oligomerization of protein was also tested as one pathway toward improved bioelectrode stability.^[^ ^110^ ^]^ Moreover, the exceptional durability of extremophilic enzymes shows significant industrial benefits, particularly in terms of their prospective biotechnological uses. Aya Kontani et al. expanded the function temperature range of biofuel cells by employing thermostable alcohol dehydrogenase: electrochemical oxidation of NADH due to enzyme‐catalyzed alcohol oxidation was obtained even at 70°C in the electrode system.^[^ ^111^ ^]^ The investigation revealed by Ganesan Sathiyanarayanan et al. shows the acidithiobacillus ferrooxidans modified electrode can work under an acidic environment even if the pH value is 2. The stability under this harsh environment is also worth mentioning, a maximum current density of −38.61 ± 13.16 A m^−2^ was obtained even after 2 weeks if the reactor was supplied with enough electron donor and iron chelator.^[^ ^112^ ^]^

Furthermore, some studies have also focused on changing the coenzyme specificity of wild type enzymes toward low‐cost alternatives by using engineered enzymes. For instance, Banta et al. modified the alcohol dehydrogenase from pyrococcus furiosus to utilize the biomimic cofactor nicotinamide mononucleotide (NMN), which offered a faster diffusion rate due to their smaller size in contrast to the natural cofactor counterparts.^[^ ^113^ ^]^ Chen et al. also altered 6‐phosphogluconate dehydrogenase's coenzyme preference from its normal cofactor. The results revealed an increase in power density and enzyme stability in a demanding environment with the engineered enzyme.^[^ ^114^ ^]^ The engineering of enzyme cofactor can also mitigate the problem associated with the natural cofactors, including high cost and sluggish diffusion kinetics. The advance in this field will significantly enhance the versatility of EBFCs associated with different types of cofactors.

## 4 NEW APPLICATIONS ENABLED BY EBFCs

In accordance with the recent trends toward net‐zero carbon emission and the need for products powered by green technology, EBFC is now being studied extensively for the applications of implantable bioelectronics, portable or wearable devices, and biosensors or detector devices. This section will present the current state of EBFCs for these applications.

### 4.1 Implantable bioelectronics

**FIGURE 9:**
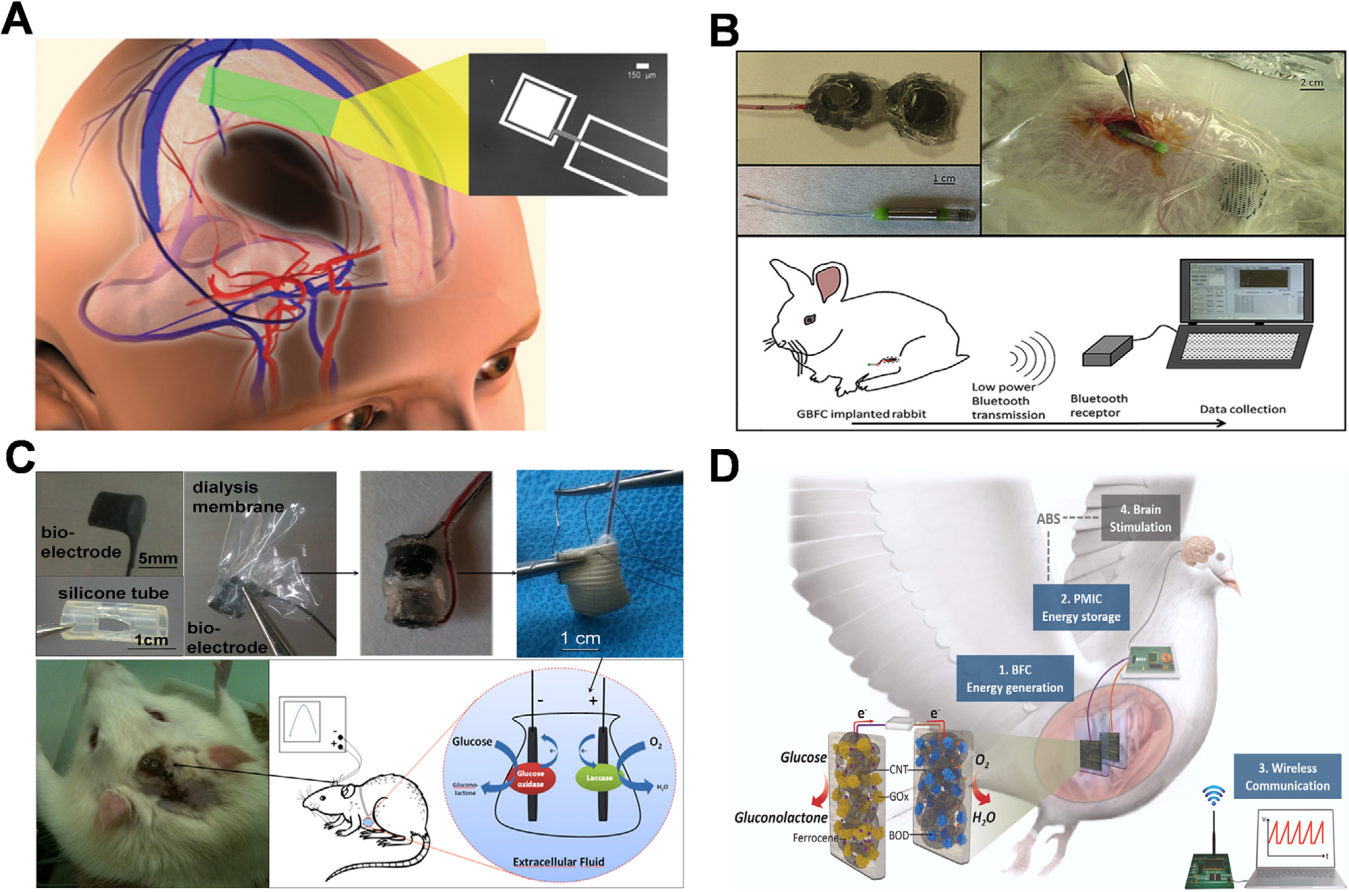
(A) Power extraction from cerebrospinal fluid by an implantable enzymatic biofuel cell. Reproduced with permission.^[^ ^119^ ^]^ Copyright 2012, Public Library of Science. (B) Enzymatic biofuel cell (EBFC) is implanted in the abdominal cavity of a rabbit. Reproduced with permission.^[^ ^116^ ^]^ Copyright 2018, Elsevier. (C) Long‐period stable EBFC‐based device implanted in a rabbit. Reproduced with permission.^[^ ^36^ ^]^ Copyright 2018, Elsevier. (D) Implantation of EBFC and animal brain stimulator in a bird. Reproduced with permission.^[^ ^117^ ^]^ Copyright 2021, Elsevier.

The use of traditional batteries in implantable devices comes with some disadvantages such as the need for battery replacement and the associated risk of triggering an inflammatory response in the body. This signifies the need for a long‐lasting and stable biofriendly energy source that can be used for implantable devices, especially for brain implantable devices (Figure 9A). Fortunately, the availability of a fuel source like glucose coupled with the physiological conditions in the body (temperature, pH) that is suitable for enzymes allows for the use of EBFC in implanted devices. Tremendous progress has been made over the past few years in translating the conceptual idea of implantable EBFCs into real applications. Following the first report by Mano and Heller of a biofuel cell implanted and operating in a living organism (grape) in 2003,^[^ ^9^ ^]^ the concept of implanted biofuel cells was further extended to amphibians, insects, crustaceans, and molluscs.^[^ ^34^, ^115^ ^]^ In particular, Katz and his colleagues reported that several EBFC‐implanted clams or lobsters can provide enough power to supply low‐power electronic products like capacitors or pacemaker.^[^ ^34^, ^115^ ^]^ While implanted EBFC have been successfully demonstrated in these creatures, the application of EBFC in mammals is also attracting research attention. Ichi‐Ribault et al. first implanted an EBFC‐based bioelectronic device connected to a tele‐transmission system into a rabbit (Figure 9B), which showed a long period of stability where the device could be monitored and controlled even for 2 months.^[^ ^116^ ^]^ Furthermore, Zebda et al. implanted an EBFC in a rat that utilizes the rat's body fluids as the sole biofuel source.^[^ ^36^ ^]^ It was shown that the implanted device's power output was sufficient to power a LED and also had high biocompatibility as there were no signs of rejection or inflammation even after 110 days of implantation (Figure 9C). Lee et al. first successfully implanted EBFC‐based animal brain stimulators into flying birds (Figure 9D). Their EBFCs’ bioanode and biocathode were assembled by utilizing GOx, and BOD respectively. These implantable EBFCs achieved continual energy supply for the animal brain stimulators over 10 min, which indicates the feasibility of constructing self‐powered neuromodulation.^[^ ^117^ ^]^ Although these reports of EBFCs operating in plants and animals are promising, the application of implanted EBFC in humans is far from completion as several hurdles including the long‐term stability of enzymes and biocompatibility still need to be addressed.^[^ ^118^ ^]^

### 4.2 Powering portable and wearable electronic devices

**FIGURE 10:**
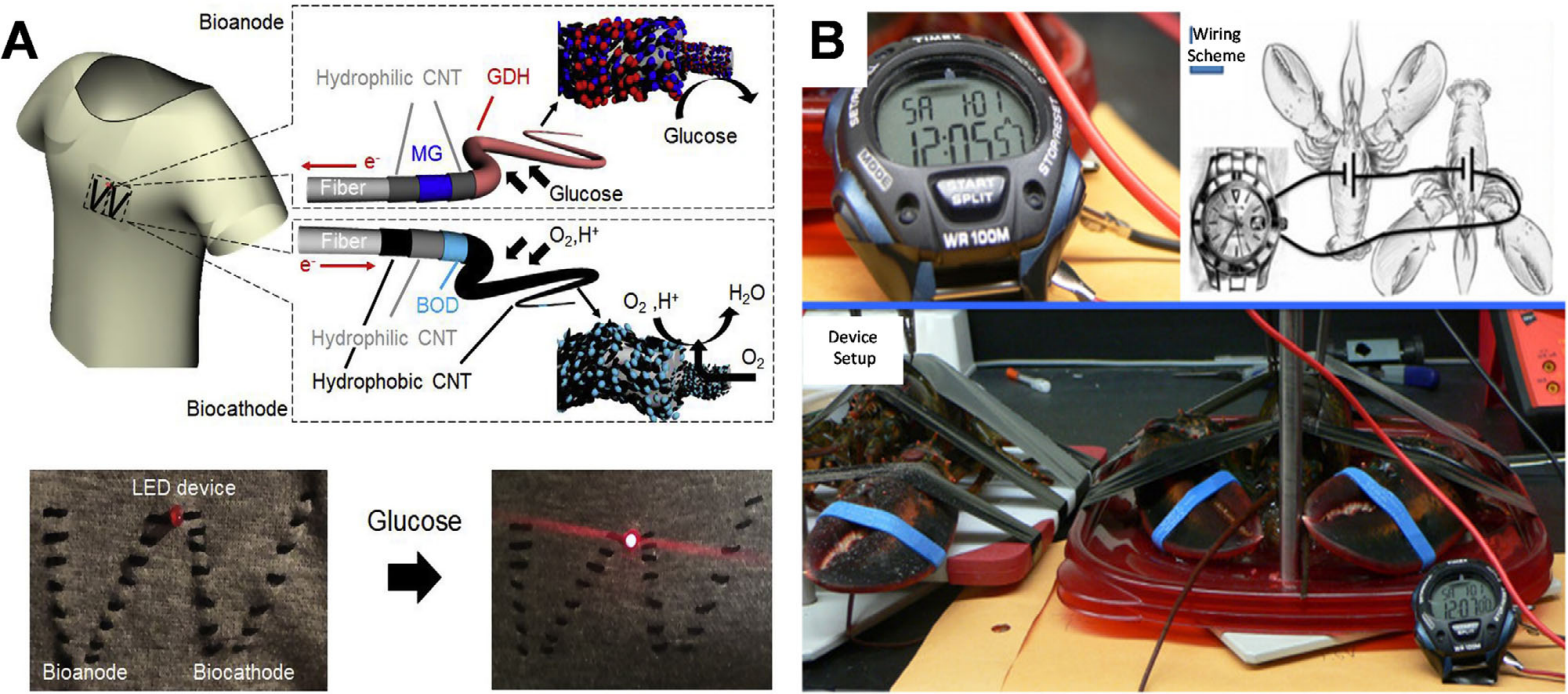
(A) Setup of enzymatic biofuel cell (EBFC) woven on a textile cloth powering up a light‐emitting diode (LED) when a glucose solution is dropped on it. Reproduced with permission.^[^ ^120b^ ^]^ Copyright 2022, Elsevier. (B) EBFC implanted in the organism as energy source for a watch. Reproduced with permission.^[^ ^122^ ^]^ Copyright 2013, Royal Society of Chemistry. GDH, glucose dehydrogenase; CNT, carbon nanotube.

Theoretical models suggest that EBFCs could achieve high energy‐storage densities if the fuel undergoes complete oxidation. In living cells, this can be accomplished through complex catabolic pathways.^[^ ^32^ ^]^ Inspired by this, Zhu et al. developed a sugar‐powered biobattery that uses a synthetic catabolic pathway consisting of 13 enzymes. With an energy‐storage density of 596 Ah kg^−1^, this biobattery surpasses the energy‐storage densities of primary batteries and lithium‐ion batteries by more than one order of magnitude.^[^ ^32^ ^]^ EBFCs have been applied as biobatteries in several studies to power light‐emitting diodes (LED),^[^ ^32^, ^45^, ^120^ ^]^ digital clocks,^[^ ^32^ ^]^ and even a music player^[^ ^121^ ^]^ by using multiple cells stacked in series. For instance, in Figure 10A, a series‐connection of cotton textile EBFC can light an LED on the fabric;^[^ ^120b^ ^]^ In Figure 10B, to generate a voltage higher than 1.0 V, two lobster‐based EBFCs were coupled to provide a voltage more than 1.0 V for powering an electrical sport watch.^[^ ^122^ ^]^ Therefore, there is a great potential for EBFCs to be used for powering portable electronics such as cellphones and laptops. Despite its promise, the implementation of EBFCs on a large scale is still hindered by potential issues related to the short lifetime of enzymes, cofactors, and mediators, which need to be resolved.^[^ ^32^ ^]^

In terms of the practical design of EBFCs for portable devices, a range of innovative microfluidic prototype designs aimed at reducing the internal resistance of the fuel cell such as 3D printed EBFCs or paper‐based EBFCs have been explored.^[^ ^123^ ^]^ Paper‐based electrical devices, in particular, show great promise because of their thin dimension, lightweight, low cost, and remarkable flexibility. Zhang et al.^[^ ^123a^ ^]^ proposed a mediator‐less and membrane‐free paper type glucose/air EBFC, which was able to work by just dripping a low‐volume solution (30 µL) containing glucose (e.g., vitamin water, fresh juice). Furthermore, Claudia et al.^[^ ^123d^ ^]^ demonstrated that a stack of paper‐based EBFCs can power a digital clock for 36 h continuously using glucose in Gatorade®, a commercial drink as fuel.

**FIGURE 11:**
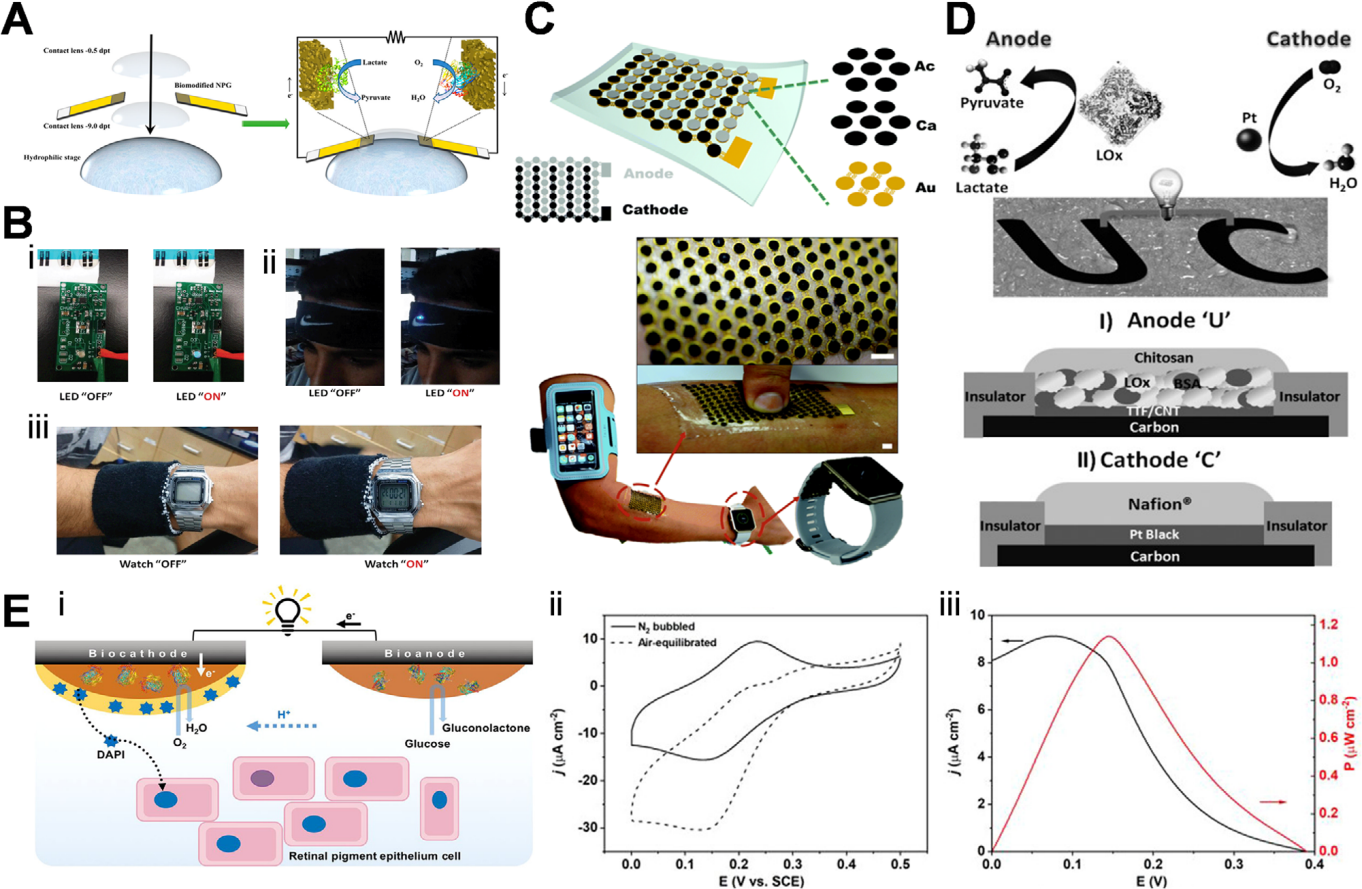
Wearable devices enabled by enzymatic biofuel cell (EBFC). (A) Contact lens encapsulated EBFC. Reproduced with permission.^[^ ^125^ ^]^ Copyright 2018, American Chemical Society. (B) Wearable textile EBFCs. Reproduced with permission.^[^ ^130^ ^]^ Copyright 2014, American Chemical Society. (C) Wearable applications coupled with a skin related EBFC. Reproduced with permission.^[^ ^127^ ^]^ Copyright 2017, Royal Society of Chemistry. (D) Illustration of the epidermal tattoo like EBFC. Reproduced with permission.^[^ ^126^ ^]^ Copyright 2013, Wiley‐VCH. (E) Scheme of EBFC‐based drug release system and profiles of CVs and power density. Reproduced with permission.^[^ ^129^ ^]^ Copyright 2020, American Chemical Society.

Among the different types of portable electronics, some researchers have also investigated the construction of EBFCs for wearable devices. Minteer and his colleagues recently integrated EBFC into a contact lens with the goal of utilizing tear to produce electricity, which exhibited an OCV around 0.413 V and a maximum current and power density of 61.3 ± 2.9 µA cm^−2^ and 8.01 ± 1.4 µW cm^−2^, respectively.^[^ ^124^ ^]^ Magner et al. further enhance the design of the EBFC contact lens by sandwiching mechanically flexible nanoporous gold electrodes between two lenses to avoid direct eye contact (Figure 11A).^[^ ^125^ ^]^ Apart from contact lenses, several studies have also been done on skin‐conformable EBFC devices (Figure 11B,C). In 2013, Wang's group prepared a tattoo‐like wearable device (Figure 11D) to harvest biochemical energy from human perspiration, which showed power densities ranging from 5 to 70 µW cm^−2^ due to variations in the lactate levels of individuals with different fitness levels.^[^ ^126^ ^]^ In a later study by the same group, a high power density of nearly 1.2 mW cm^−2^ at 0.2 V was achieved for skin‐based EBFC, which can successfully power a Bluetooth low energy radio.^[^ ^127^ ^]^ Apart from skin‐conformable EBFC, Wang et al. also integrated a six‐stack biofuel cell into a bandage and sportswear, which could harness enough energy from sweat to power up a sports watch.^[^ ^128^ ^]^ Very recently, Xiao et al. achieved an EBFC‐based drug delivery system that exhibited potential for transdermal drug permeation as shown in Figure 11E.^[^ ^129^ ^]^ Their EBFC has a pharmacological layer and can be activated by glucose and oxygen. In the presence of biofuels, EBFC starts to release drugs. Three different types of drugs’ successful release demonstrate their system is proof‐of‐concept and may be utilized in wearable or even implantable medication delivery devices. Although these examples of wearable EFBC are promising, the development of an efficient, long‐lasting EFBC wearable device still needs to be further explored.

### 4.3 Self‐powered biosensor and pollutant detector

Another potential application of EBFC is for self‐powered biosensors. Conventionally, a biosensor is a transducer or electrode coated with a layer of selective receptor containing specific biological entities (protein or enzyme, nucleic acid, or even bacteria). This device usually requires an external power source to drive its operation. In the case of a self‐powered biosensor, EBFC is used as the energy source and the cell output signal is used as an analysis and detection signal to achieve analyte quantification.^[^ ^131^ ^]^ This ingenious assembly was first introduced by Katz et al.^[^ ^131^ ^]^ in 2001, which used glucose or lactate as the analyte/fuel. Li's group^[^ ^132^ ^]^ later constructed a self‐powered homogeneous immunosensing platform for the ultrasensitive detection of melamine in milk through the strategy of target‐induced biofuel release. Recently, an EBFC‐based wearable biosensor (diaper) was also reported by Zhang et al.^[^ ^133^ ^]^ The wearable biosensor utilized glucose in urine as biofuel to drive the flashing of a LED, where the power output of the EFBC was reflected by the frequency of flashes for the subsequent determination of glucose concentration in urine.^[^ ^133^ ^]^ Furthermore, a self‐powered cytosensor has also been constructed by Gai et al. for the purpose of disease diagnosis, specifically for the detection of acute leukemia CCRF‐CEM cells.^[^ ^134^ ^]^ In this work, CCRF‐CEM cells can be captured by the cathode via aptamer recognition, which will block the electron transfer between the probe and cathode and cause a drop in power output, thereby allowing the detection of the cells.^[^ ^134^ ^]^

Aside from biomass detection, self‐powered EBFC devices can also be utilized for pollutant detection by employing different kinds of enzymes. For instance, laccase can be used in a self‐powered biosensor for BPA detection. The laccase‐based single enzyme biofuel cell was first introduced by our group to generate electricity, and at the same time degrade micropollutants such as BPA by utilizing it as the fuel. In a subsequent study conducted by Li et al.,^[^ ^63^ ^]^ it was shown that it could also function as a self‐powered biosensor for BPA detection with a detection limit of 1.95 × 10^−3^ mm.

## 5 CHALLENGES

Since the creation of the EBFC, this technology has faced numerous obstacles that can be classified into three categories: stability‐based challenges, performance‐based challenges, and compatibility‐based challenges. Decades of continual study in EBFC have produced a variety of solutions to these issues, while more efficient solutions to these challenges are still highly sought after in order to consistently produce more efficient and resilient EBFC.^[^ ^135^ ^]^ Stability‐based challenges revolve around the EBFC's ability to sustain itself in a robust environment over time, whereas performance‐based challenges revolve around the EBFC's ability to generate enough useful output for its desired application. Compatibility‐based challenges are more focused on specific applications, in which the EBFC's compatibility with the environment/host should be considered. While the research to improve the EBFC and address these concerns has been continuing, several of these issues remain unresolved. This section aims to provide a quick overview of the previous and current challenges faced by EBFC, offering some guidelines for the pressing problems in this field.^[^ ^8^, ^12^, ^136^ ^]^

### 5.1 Stability‐based challenges

For an EBFC to be beneficial, it must be able to sustain its performance over a prolonged length of time. Stability concerns in EBFC can be caused by either enzyme stability or electrode stability issues. Enzyme‐related stability issues are almost identical to those encountered in other enzyme‐containing systems, as enzymes are known to be sensitive to their surroundings, particularly pH and temperature.^[^ ^137^ ^]^ Enzymes typically perform best within their optimal pH and temperature ranges and can easily denature when utilized outside of their optimal pH and temperature ranges. Denaturation of these enzymes at high temperatures or pH levels is often irreversible, rendering the entire EBFC useless. The enzyme may also detach from the electrode over time, resulting in a drop in electron production and, as a result, an irreversible loss in EBFC performance.^[^ ^138^ ^]^ To overcome this, researchers have devised several solutions such as enzyme engineering and enzyme immobilization, which are discussed in Section 3.2. However, engineering an enzyme system that is resistant to a greater range of temperature and pH over an extended length of time remains a difficulty. EBFC systems have been shown to last up to 2 months in the past, although most EBFC systems today last far less than that under continuous usage.^[^ ^139^ ^]^ A lengthier stability test should be carried out to ensure that the EBFC's integrity as a power supply for various devices can be maintained throughout a prolonged useful lifetime. This will be especially crucial for systems that cannot replace their power source as frequently. For example, a longer‐lasting power source—preferably lasting the lifespan of the host—will be most desirable for usage as a power source in pacemakers, which will result in a costly and risky operation to replace.^[^ ^140^ ^]^

Another stability‐related challenge concerns the electrode's stability. Due to the nature of the surrounding biofuel in the EBFC's environment, EBFCs can be prone to biofouling. Proteins and other biological molecules can deposit on the electrode of the EBFC, resulting in a more inefficient pathway for biofuels to reach the active sites of the enzymes in the EBFC, dramatically lowering the EBFC's efficiency and energy output over time.^[^ ^141^ ^]^ Researchers have devised a solution to this problem by adding anti‐biofouling coatings onto EBFC electrodes to limit the influence of biofouling, but a more in‐depth and longer stability test should be undertaken to completely understand the biofouling effect on EBFC over a longer period.^[^ ^141c^ ^]^

### 5.2 Performance‐based challenges

Aside from its capacity to maintain optimal performance over an extended length of time, the EBFC must also create enough output to be useful for its intended applications. This presents additional issues for the EBFC in terms of generating enough energy, power, and voltage to power the device.

The energy produced by contemporary EBFC is typically minimal, with a range of only a few mW.^[^ ^139a^ ^]^ Although these energy ranges are adequate for some applications, such as implantable medical devices and biosensors, an increase in energy generated within future EBFC systems will undoubtedly increase the efficiency and usage of current EBFC‐containing devices, while also allowing EBFC to be used in other more energy‐intensive applications. As naturally occurring enzymes are known to create a minimal quantity of energy, greater optimization toward improving enzyme activity will undoubtedly be the task to be tackled in order to remedy this issue.^[^ ^12a^ ^]^ Developing strategies to increase enzyme activity, such as using an enzymatic cascade system or enzyme engineering, using highly porous and conductive nanomaterials as electrodes to help increase surface to volume ratio for higher enzyme loading, and improving the mass transfer of fuel and product via methods such as gas diffusion bioelectrodes and microfluidic EBFC systems are currently being investigated to improve energy density in EBFC.^[^ ^4^, ^142^ ^]^

**FIGURE 12:**
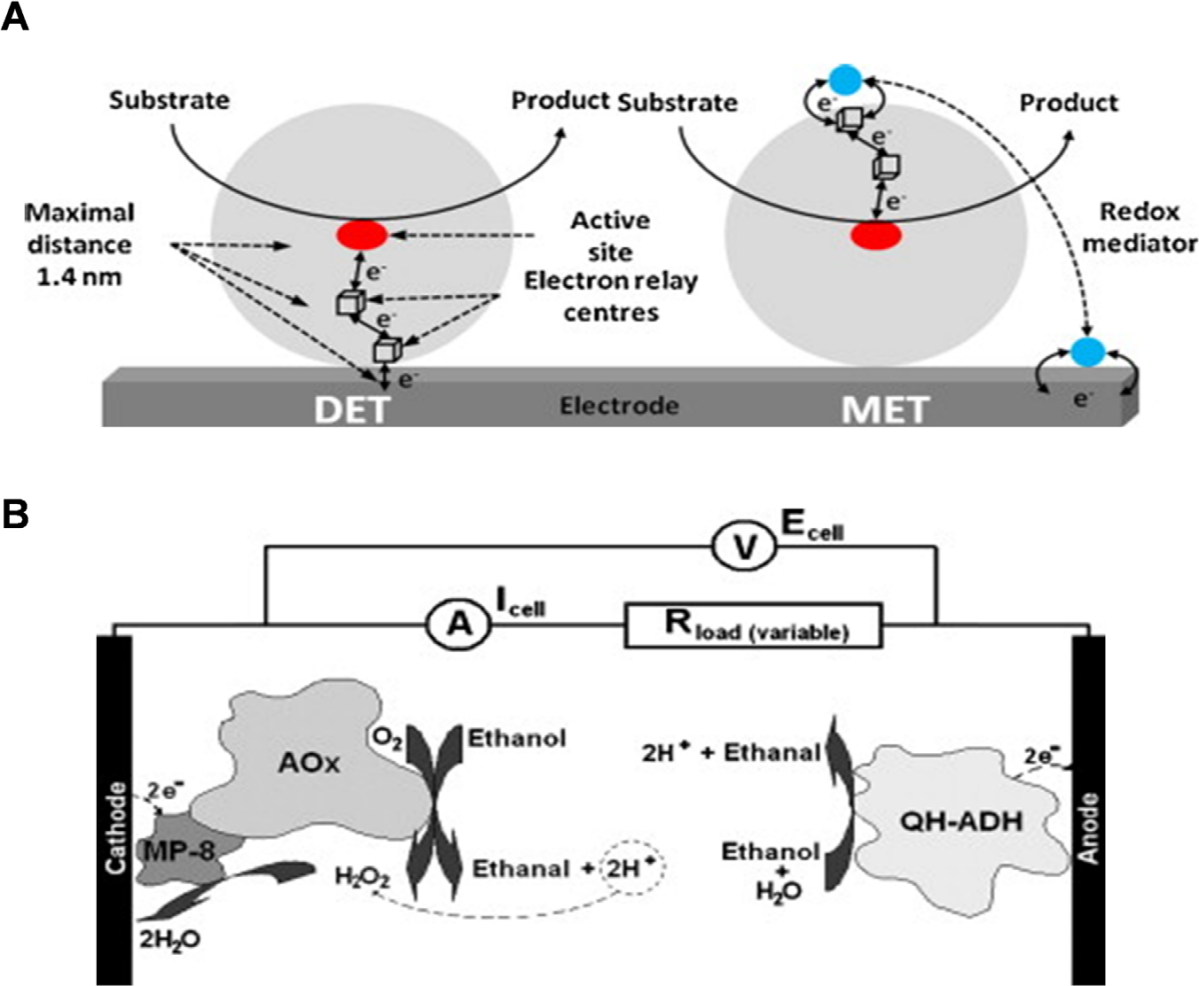
(A) Schematic representation of direct electron transfer (DET) and mediated electron transfer (MET). Reproduced with permission.^[^ ^147^ ^]^ Copyright 2014, Elsevier. (B) Configuration of enzymatic biofuel cell utilizing ethanol as fuel for both anode and cathode. Reproduced with permission.^[^ ^145a^ ^]^ Copyright 2008, Elsevier.

The transmission of electrons from the enzyme to the electrode is also a difficulty for a higher energy density EBFC. Poor electron transmission from the enzyme to the electrode could create a bottleneck in the EBFC's total output, restricting its use. In EBFC, electron transfer can occur via two main pathways: direct electron transfer (DET) and mediated electron transfer (MET), as illustrated in Figure 12A.^[^ ^143^ ^]^ DET is a very useful pathway that involves the direct transfer of electrons from the enzyme to the electrode. For example, Ramanavicius et al. immobilized quino‐hemoprotein‐alcohol dehydrogenase (QH‐ADH) onto a carbon rod electrode, which was able to directly generate electric potential with a maximal open circuit potential of −115 mV.^[^ ^144^ ^]^ In another study, a biofuel cell in which the electrodes were based on three kinds of enzymes was also investigated as shown in Figure 12B, it utilized the same type of fuel at both electrodes (ethanol) and thus avoided the need for a compartmentalized biofuel cell.^[^ ^145^ ^]^ Nevertheless, it should be noted that the majority of enzymes are not able to transfer electrons to the electrode directly.^[^ ^144a^ ^]^ Therefore, another pathway (MET pathway) that involves the use of a redox mediator is necessary to aid the transfer of electrons from the enzyme to the electrode. DET should theoretically offer a higher power density than MET, however past study has proved otherwise.^[^ ^146^ ^]^ As a result, it is an engineering issue to ensure that the full potential of DET is leveraged in order to improve power generation in EBFC. Because of the addition of mediator chemicals, MET systems may impose extra compatibility and stability issues for specific applications, which will be explored in a later section.

Aside from energy density, it is also critical for EBFC to constantly provide enough voltage to power particular devices. Current commercially available microdevices typically operate on voltages ranging from 1–3 V.^[^ ^34^ ^]^ In comparison, EBFC usually generates much lower voltage. Ideally, the maximum voltage glucose‐based EBFC can generate is around 1.18 V with two‐electron oxidation of the fuel.^[^ ^12^, ^34^ ^]^ Some strategies that have been explored to improve the voltage in EBFC include employing serial connection circuit in EBFC powered devices and employing an external boost converter or voltage amplifier.^[^ ^148^ ^]^ MET systems may also provide an increase in voltage output over its DET counterpart as the presence of a redox mediator has been shown to be able to provide a lower energy barrier to overcome.^[^ ^149^ ^]^ However, this method still requires an extensive optimization for each unique enzymatic system to ensure the compatibility between the redox mediator and enzyme can provide the highest possible voltage.^[^ ^150^ ^]^

### 5.3 Compatibility‐based challenges

Compatibility‐based difficulties also play a role in ensuring that the appropriate EBFC is built for certain applications. This includes verifying that the EBFC system is appropriate for the environment in which it will operate and will not harm the environment. When enhancing EBFC or building new more efficient EBFC for specific usage, it is always vital to consider the host system's compatibility. For example, using an enzyme cascade to generate energy by converting CO_2_ to methanol will almost surely aid boost electron generation in an EBFC that employs this enzymatic cascade.^[^ ^12a^ ^]^ This technique, however, will not be ideal for implantable medical devices or wearable sensors since it produces harmful byproducts such as formic acid and methanol. This difficulty also exists in MET‐based EBFC, since the redox mediator in the MET‐based EBFC system can either leak from the electrode or be abundant in the environment surrounding the electrode.^[^ ^12^, ^146^ ^]^ As a result, the toxicity of the redox mediator toward the environment/host for these EBFC‐based devices poses a significant problem that must be addressed.

## 6 CONCLUSION AND FUTURE PROSPECT

**FIGURE 13:**
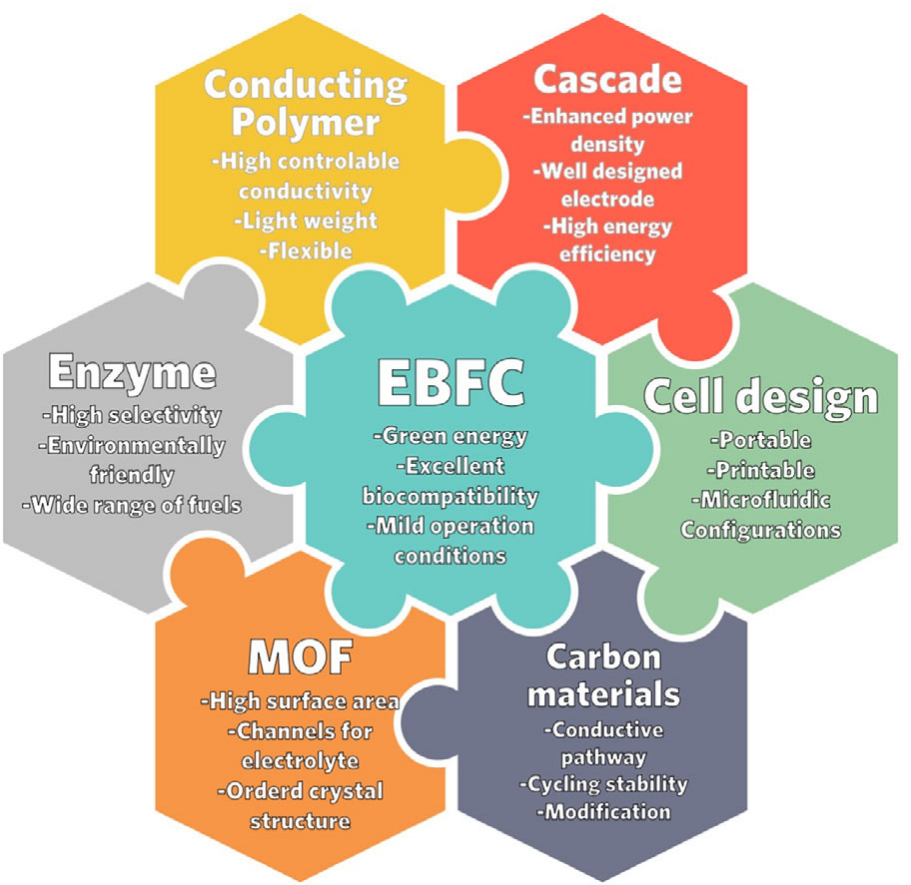
Schematic illustration of the strategic areas for improving enzymatic biofuel cell (EBFC) performance.

Renewable energy generation has always been an appealing field to investigate as a long‐term ecologically sustainable energy solution. EBFC is a promising green energy generation device that derives its electricity from renewable biofuels while also being biodegradable. The enzymes, which are the primary energy generators in EBFC, are typically derived from renewable sources as well and exhibit great advantages over synthetic catalysts in conventional fuel cells in terms of reaction rates and selectivity. However, according to current research, there is still a large gap between EBFCs and commercially available chemical fuel cells, especially in terms of energy density and operational lifetime. Extensive research has been conducted to improve the operational stability and enhance the energy output density of the EBFC. Numerous promising directions for addressing these challenges have been and continue to be explored, including novel cell designing, fabrication of advanced materials, complete oxidation of fuel, introduction of novel redox enzymes like extremozymes, identification of redox centers and technologies for enzyme design, as depicted schematically in Figure 13.

### 6.1 Optimal electrode design

**FIGURE 14:**
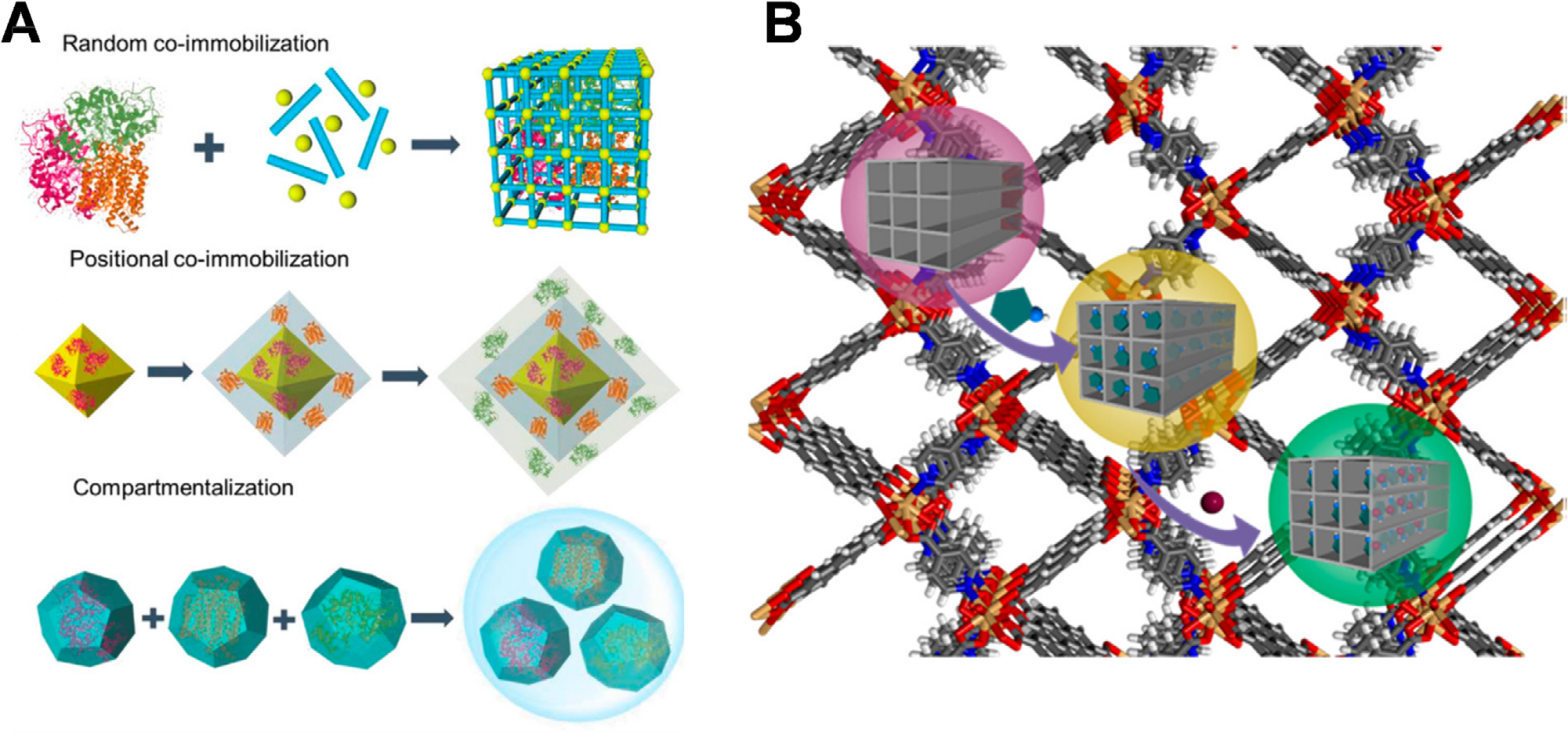
(A) Schematic of immobilization strategies of enzyme cascade inside metal‐organic framework (MOF). Reproduced with permission.^[^ ^153^ ^]^ Copyright 2020, Wiley‐VCH. (B) Schematic of incorporation of conducting polymer (CP) inside MOF. Reproduced with permission.^[^ ^154^ ^]^ Copyright 2016, American Chemical Society.

The main method for enhancing EBFC's performance is to achieve complete oxidation of the fuel. As mentioned in Section 2.3.1, enzyme cascades have shown promising potential for improving power density through a more complete fuel oxidation. Moreover, well designed enzyme cascade integrated in electrodes can harvest more energy while achieving enzyme cofactor regeneration. However, the complexity of multienzyme‐based electrode usually results in poor stability or even ineffective enzyme immobilization. The intermarriage between biology and materials engineering will be required to enable the design and application of more efficient cascade EBFC with high stability and specific activity. For instance, hierarchical three‐dimensional or layer‐by‐layer bioelectrode scaffolds can achieve much higher power density when used for enzyme immobilization on an electrode due to precise control of the degree of dimensionality and orientation of the material. Three‐dimensional graphene or hierarchical mesoporous MOF (as shown in Figure 14A) can entrap more enzymes and mitigate enzyme leaching. Their bio‐compatibility further promotes their use as a versatile platform for cascade EBFC.^[^ ^151^ ^]^ Layer‐by‐layer assembly of bio‐electrode can also enable adjustable immobilization of different types of enzymes by constructing an easily operable multilayer structure, which exhibits good prospects in cascade EBFC.^[^ ^152^ ^]^ Owing to the unique properties of these bioelectrode scaffolds, we consider that reliable reporting of these related nanomaterials will benefit the oxidation of fuel and further improve its power density.

An alternative method for enhancing EBFC's performance is to create new designs of bio‐electrodes with high surface areas and good electrical properties in order to improve the electron's charge and transfer process at the enzyme‐electrode interface and to optimize the pore structures of the porous matrix for efficient mass transport. For example, the combination between MOF materials and conducting polymer (CP) is highly promising owing to MOF's high surface area and CP's good conductivity. It has always been challenging for MOF materials to achieve effective electron separation and transfer, although MOFs exhibit remarkable pore volume and well‐defined structure for enzyme protection. Redox polymer or some conducting polymer like polypyrrole (PPy), polyaniline, and poly(3,4‐ethylenedioxythiophene) (PEDOT) have been extensively studied for its high, stable, and tunable electrical conductivity.^[^ ^155^ ^]^ Recently, it has been demonstrated that the incorporation of PPy into MOFs produces electrode materials with remarkable electrochemical performance (as shown in Figure 14B).^[^ ^154^ ^]^ Inspired by the intermarriage between MOF and CP, we consider the combination of conductivity and porosity in EBFC's bio‐electrode will endow EBFC with unique possibilities: large and electrochemically active surface area with redox‐active sites and enough conductive pathways for a swift charge transport.

### 6.2 Novel cell design

**FIGURE 15:**
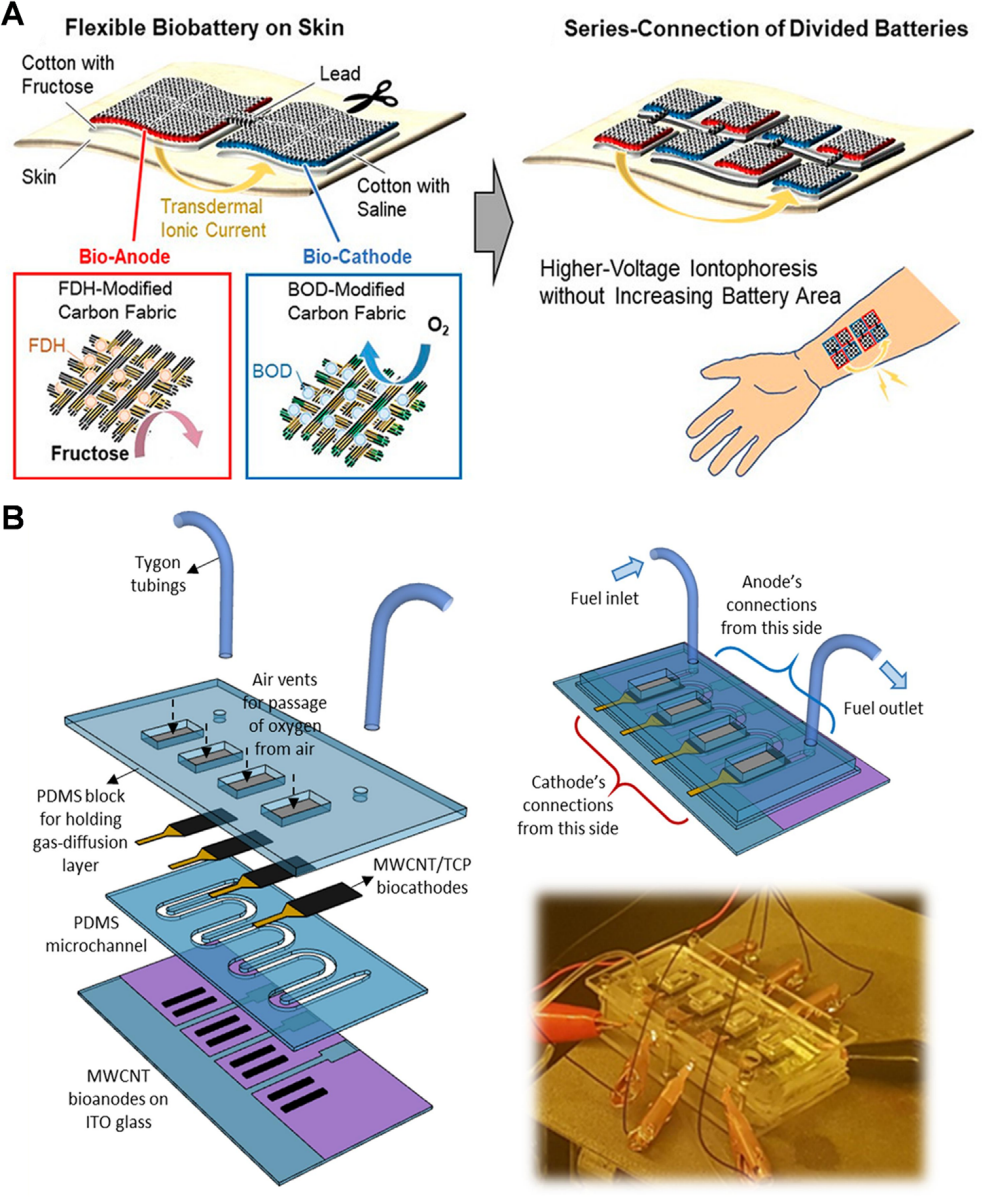
(A) Construct a series of flexible enzymatic biofuel cells (EBFCs) to obtain higher voltage. Reproduced with permission.^[^ ^156^ ^]^ Copyright 2019, American Chemical Society. (B) Illustration of microfluidic EBFCs stacking device components and the product image. Reproduced with permission.^[^ ^157^ ^]^ Copyright 2022, Elsevier. FDH, fructose dehydrogenase; BOD, bilirubin oxidase; MWCNT, multi‐walled carbon nanotube.

The initial achievements in self‐powered electronics, as mentioned in Section 4, encourage the employment of EBFCs in medical applications or wearable devices including pacemakers, cancer detection, and paper‐based devices. The self‐powered EBFC implantable electronics are still primarily used in vitro due to the fact that they are made with rigid and large‐scale electrodes, which can seriously clog blood vessels, despite the fact that the scale of EBFC and the use of exogenous biomaterials, which have negative effects on the human body, have been reduced and eliminated, respectively. Therefore, efforts should be made to integrate mature biocompatible microelectrodes with the novel EBFC cell design, thereby promoting the deployment of implanted devices or portable diagnostic devices. As shown in Figure 15A, tiny flexible EBFC can be fabricated separately, and then connected to maintain a higher voltage.^[^ ^156^ ^]^ A similar strategy could be adopted for in vivo EBFC‐based device fabrication.

Since microfluidic EBFCs can be included in a range of bio‐devices, they are attracting an increasing amount of research interest.^[^ ^158^ ^]^ Benefits such as a high reaction rate and mass transfer at a miniature scale enable microfluidic EBFCs‐based devices to minimize response times and costs. Meanwhile, it also exhibited the ability to achieve high power density and voltage. As shown in Figure 15B, a novel stacking of single‐stream microfluidic EBFCs exhibited enhanced practicality.^[^ ^157^ ^]^ Besides, paper‐based EBFC, an example of microfluidic EBFCs in which the mass transport is based on capillary action, can realize laminar flow and there is no mixing between fuel and oxidant.^[^ ^159^ ^]^ Moreover, the capillary action does not rely on external pressure sources and thus can work in vivo. Similarly, 3D‐printing or flexible EBFC‐based miniaturized devices are also reported.^[^ ^160^ ^]^ In vivo applications based on related microfluidic cell protypes have been developed such as self‐powered glucose biosensor and cancer diagnosis device, which can pave the way for more advanced in vivo EBFC‐based devices.

All in all, the introduction of advanced materials allows the EBFC to exhibit excellent electrical and mechanical properties.^[^ ^161^ ^]^ The successful integration of advanced biomaterials with unique cell design also extends EBFC's application.^[^ ^162^ ^]^ Because of its high biocompatibility, EBFC has potential to be used in implantable medical devices and portable bioelectronic/biosensor devices. Although there are still obstacles to overcome before the EBFC can be used in other applications such as large‐scale energy generation, the existing EBFC technology is clearly promising and can already be applied to tackle lab‐scale issues, specifically in medical‐related areas. The translation of EBFCs from bioelectrochemical systems and laboratory prototypes to viable technology still relies on the interdisciplinary investigation, which involves biology, chemistry, materials and chemical engineering, and even medical science. Admittedly, there are unique advantages of the EBFC operation over the other energy resources. Although there is still more scope for improvement, we are optimistic that future advances in this field will assist its commercialization.

## CONFLICT OF INTEREST STATEMENT

The authors declare no conflicts of interest.
